# HY5 functions as a systemic signal by integrating BRC1-dependent hormone signaling in tomato bud outgrowth

**DOI:** 10.1073/pnas.2301879120

**Published:** 2023-04-10

**Authors:** Han Dong, Jiachun Wang, Xuewei Song, Chaoyi Hu, Changan Zhu, Ting Sun, Zhiwen Zhou, Zhangjian Hu, Xiaojian Xia, Jie Zhou, Kai Shi, Yanhong Zhou, Christine H. Foyer, Jingquan Yu

**Affiliations:** ^a^Department of Horticulture, Zijinggang Campus, Zhejiang University, Hangzhou 310058, China; ^b^College of Horticulture, Northwest Agriculture & Forestry University, Xianyang, Shaanxi 712100, China; ^c^School of Biosciences, College of Life and Environmental Sciences, University of Birmingham, B15 2TT Edgbaston, UK; ^d^Key Laboratory of Horticultural Plants Growth and Development, Agricultural Ministry of China, Zhejiang University, Hangzhou 310058, China

**Keywords:** bud outgrowth, BRANCHED1, phytohormone, light signal, plant architecture

## Abstract

Light plays an important role in determining plant architecture, which greatly influences crop yield. However, the precise mechanisms by which light signaling regulates bud outgrowth remain to be identified. Here, we show that light regulates bud outgrowth via both HY5 and brassinosteroid (BR)-dependent pathways in tomato. Inactivation of the red-light photoreceptor PHYB, or deficiencies in PHYB or the blue-light photoreceptor CRY1a, inhibits bud outgrowth and leads to decreased accumulation of HY5 protein and increased transcript level of *BRANCHED1* (*BRC1*), a central integrator of branching signals. HY5, functioning as a mobile systemic signal from leaves, promotes bud outgrowth by directly suppressing *BRC1* transcript and activating the transcript of BR biosynthesis genes within the lateral buds in tomato. Furthermore, BRC1 prevents the accumulation of cytokinin (CK) and gibberellin (GA) by directly inhibiting the transcript of CK synthesis gene *LOG4*, while increasing the transcript levels of CK and GA degradation genes (*CKX7*, *GA2ox4*, and *GA2ox5*), leading to an arrest of bud outgrowth. Moreover, bud outgrowth occurs predominantly in the day due to the suppression of *BRC1* transcript by HY5. These findings demonstrate that light-inducible HY5 acts as a systemic signaling factor in fine-tuning the bud outgrowth of tomato.

## Significance

The orchestration of shoot architecture, which is a major determinant of crop productivity, requires the regulated suppression or activation of -bud outgrowth. We show that light quality regulated tomato bud outgrowth is dependent on the mobile signaling protein HY5 and that HY5 dependent light signaling alone is sufficient to regulate bud outgrowth. HY5 promotes bud growth by direct and brassinosteroid mediated suppression of BRC1. BRC1 blocks the accumulation of cytokinin and gibberellin that regulate bud growth in tomato. In this way, HY5 represses gibberellin mediated stem elongation during photomorphogenesis while activating branching through BRC1 dependent phytohormone regulation. The HY5–BRC1 module thus plays predominant roles in the shoot architecture by orchestrating light quality dependent changes in stem elongation and bud outgrowth.

Shoot architecture is an important agronomic trait that greatly affects the yield and quality of agricultural and horticultural crops (1–4). It has a great influence on light capture, photosynthesis, and resource allocation (5, 6). While appropriate branching and tillering traits are crucial to crops such as rice and wheat, the maintenance of apical dominance is important in crops such as tomato and cucumber, not least because the labor-intensive removal of excess branches is essential to achieve high yields. Shoot branching is dependent on the formation of axillary buds in the leaf axils, as well as subsequent outgrowth or dormancy. Formation of axillary buds happens largely independently of environmental conditions, whereas bud outgrowth displays a high level of plasticity in response to changes in environment conditions (5, 7–9).

Light is not only a major source of energy but also provides crucial environmental signals that control bud outgrowth. Light signals are perceived by several types of photoreceptors including the plant phytochrome, cryptochrome, and phototropin proteins. Photoreceptors regulate photomorphogenesis via the direct inhibition of transcription factors such as the phytochrome-interacting factors, as well as suppression of the functions of the COP1/SPA E3 ligase complex with the associated induction of LONG HYPOCOTYL 5 (HY5) (10, 11). Deficiency in the red-light photoreceptor PHYB or inactivation of PHYB by low red/far-red (R/FR) light ratios impairs bud outgrowth (12–16). However, the downstream components in the photoreceptor-mediated pathways that control bud outgrowth remain to be identified. Furthermore, few studies have addressed the question of the sites of light perception that regulate bud outgrowth, and the role of leaves in the bud outgrowth was largely ignored.

Plant hormones especially auxin, cytokinins (CKs), strigolactones (SLs), and sugars play a role in the regulation of axillary bud growth. Bud outgrowth is inhibited by auxin and SLs but promoted by CKs and sugars (17–19). Previously, we have demonstrated that brassinosteroid signaling integrates multiple pathways that control shoot branching by the direct transcriptional regulation of the master repressor of branching, BRANCHED1 (BRC1), through the BR signaling component BRASSINAZOLE RESISTANT1 (BZR1) in tomato (20). However, to date, information concerning the role of light quality on these hormones has remained fragmentary. Bud outgrowth is shown to be promoted by suppression of the auxin signal and induction of CK signaling by PHYB (21, 22) as well as by the transcriptional inhibition of the SL biosynthesis- and signaling-related genes by high R/FR light ratios (12, 23, 24). In addition, the bud outgrowth response to high R/FR ratios is negatively correlated with abscisic acid (ABA) levels and to the expression of ABA biosynthesis- and signaling-related genes in several species (25–28). Such findings raise the question whether the light regulation of bud outgrowth is dependent on BR signaling.

The TCP transcription factor BRC1 in dicotyledonous plants and its homolog TB1 in monocotyledonous species are specifically expressed in lateral buds. The levels of *BRC1* transcripts are regulated by hormones and sucrose (29, 30). The BRC1 protein also binds directly to the promoters of several genes involved in ABA signaling and auxin transport, thereby inhibiting the outgrowth of lateral buds in *Arabidopsis* and cucumber, respectively (1, 31). However, relatively little information is available concerning how BRC1 mediates light-regulated bud outgrowth.

Here, we show that light-regulated bud outgrowth is HY5- and BZR1-dependent in tomato. Red light or blue light induces an increase in the abundance of HY5 in the leaves. By moving from the leaves, HY5 directly suppresses the expression of *BRC1* but activates the expression of BR biosynthesis genes in lateral buds. BRC1 suppresses bud outgrowth by directly repressing the transcription of the CK biosynthesis gene *LOG4*, while simultaneously activating the expression of the CK degradation gene *CKX7* and the GA degradation genes *GA2ox4* and *GA2ox5* in tomato. Together, these changes lead to decreases in CK and GA levels and the arrest of bud outgrowth in tomato. These findings not only deepen our understanding on the light regulation of lateral organ development, but also lay the foundations for optimizing plant architecture by light manipulation of plant development, and thus increasing crop yield and quality.

## Results

### Light Regulates Bud Outgrowth via BR-Dependent and -Independent Pathways in Tomato.

**Fig. 1.**
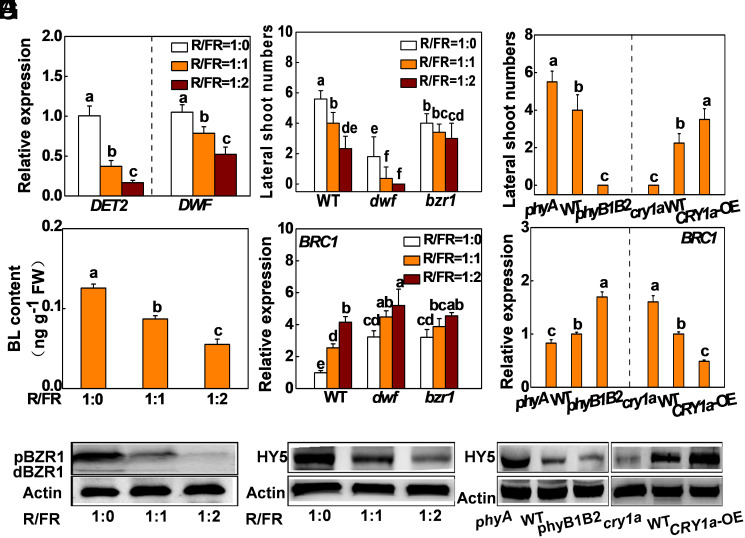
Light regulates bud outgrowth via (BR)-dependent and -independent pathways in tomato. (*A*–*C*) Transcript of *DET2* and *DWF*, BL level, and accumulation of BZR1 protein in the lateral buds in WT plants after exposure to different light regimes of R/FR ratio. (*D* and *E*) Bud outgrowth and transcript of *BRC1* in the lateral buds of BR mutants after exposure to different light regimes of R/FR ratio. (*F* and *G*) Bud outgrowth and transcript of *BRC1* in the lateral buds of photoreceptor mutants, WT and *CRY1a*-OE plants under WL. (*H* and *I*) The accumulation of HY5 protein in the lateral buds in WT plants after exposure to different light regimes of R/FR ratio and in the lateral buds of photoreceptor mutants, WT and  *CRY1a*-OE plants under WL. Samples were taken at 12 AM in *G* and *I*, or 6 h after the light quality treatment in *A*–*C*, *E*, and *H*. *ACTIN2* and *UBI3* were used as reference genes with the expression in WT as 1. Actin was used as a loading control for the western blot analysis. Data are presented as the means of replicates ± SD. n = 12 to 15 in D and F and n = 3 to 4 in *A*, *B*, *E*, and *G*. Different letters indicate significant differences (*P *< 0.05) according to Tukey’s test.

We have previously reported that BR signaling integrates multiple hormone and sugar pathways to release apical dominance in tomato (20). To determine whether BR mediates the light regulation of bud outgrowth, wild-type (WT) plants at the eight-leaf stage were placed under three light-quality regimes with red/far-red (R/FR) ratios of 1:0; 1:1, or 1:2, respectively. Decreasing the R/FR ratios in the growth environment significantly increased the levels of *BRC1* transcripts and suppressed bud outgrowth as indicated by the decreased number and total length of the lateral shoots in WT plant (*SI Appendix*, Fig. S1). The suppression of bud outgrowth by FR was associated with the lower levels of transcripts encoding the BR synthesis genes (*DET2* and *DWF*), lower accumulation of brassinolide (BL, the main active BR), and amounts of the BZR1 protein (particularly the dephosphorylated active form of BZR1, dBZR1) in the lateral buds (Fig. 1 *A*–*C*).

To explore the role of BR synthesis and signaling in light-regulated bud outgrowth, WT plants, *dwf* mutants that are defective in the BR biosynthesis gene *DWF*, and *bzr1* mutants that are defective in the BR signaling component *BRASSINAZOLE RESISTANT1* (*BZR1*) at eight-leaf stage were exposed to different R/FR light ratios (i.e., 1:0; 1:1, and 1:2, respectively). Low R/FR light ratios suppressed bud outgrowth and increased the levels of *BRC1* transcripts not only in WT plants, but also in the *dwf* mutants and *bzr1* mutants although the effects were less significant than those in the WT (Fig. 1 *D* and *E* and *SI Appendix*, Fig. S2 *A* and *B*). These results suggest that both BR-dependent and BR-independent signaling pathways are involved in the light regulation of bud outgrowth in tomato.

Next, we examined whether light signaling directly regulates bud outgrowth. *phyA* (the FR light photoreceptor mutant) had increased number and total length of the lateral shoots, and lower levels of *BRC1* transcripts under white light (WL). The double mutants *phyB1B2* (the R light photoreceptors mutant) had decreased number and total length of the lateral shoots, together with increased levels of *BRC1* transcripts (Fig. 1 *F* and *G* and *SI Appendix*, Fig. S3 *A* and *B*). In addition, the blue-light photoreceptor mutant *cry1a* had significantly decreased number and total length of the lateral shoots compared to the WT. In contrast, overexpression of *CRY1a* (*CRY1a*-OE) significantly promoted bud outgrowth. In agreement with this finding, the levels of *BRC1* transcripts were significantly higher in the *cry1a* mutants and lower in the *CRY1a*-OE plants than those of WT (Fig. 1 *F* and *G* and *SI Appendix*, Fig. S3 *A* and *B*). This suggests that these photoreceptors actively participate in the regulation of bud outgrowth, which is closely related to the changes in *BRC1* transcript levels in the lateral buds in tomato.

### HY5 Integrates Light Signaling to Regulate Bud Outgrowth in Tomato.

HY5, a basic leucine zipper transcription factor, acts downstream of various photoreceptors and participates in multiple processes that regulate plant growth and metabolism (32–34). To investigate whether HY5 plays a role in bud outgrowth, we examined the response of *HY5* transcripts and HY5 protein accumulation in buds under different light conditions. The levels of *HY5* transcripts and the accumulation of HY5 protein were decreased in the lateral buds of the WT plants when the R/FR ratios were decreased (Fig. 1*H* and *SI Appendix*, Fig. S2*C*). Moreover, compared to the WT, the accumulation of *HY5* transcripts and HY5 protein was lower in the lateral buds of the *phyB1B2* mutants but increased in the *phyA* lateral buds under WL (Fig. 1*I* and *SI Appendix*, Fig. S3*C*). In addition, the accumulation of *HY5* transcripts and HY5 proteins was lower in the buds of the *cry1a* mutant than that of the WT and increased in the *CRY1a*-OE buds compared to the WT plants (Fig. 1*I* and *SI Appendix*, Fig. S3*C*).

**Fig. 2.**
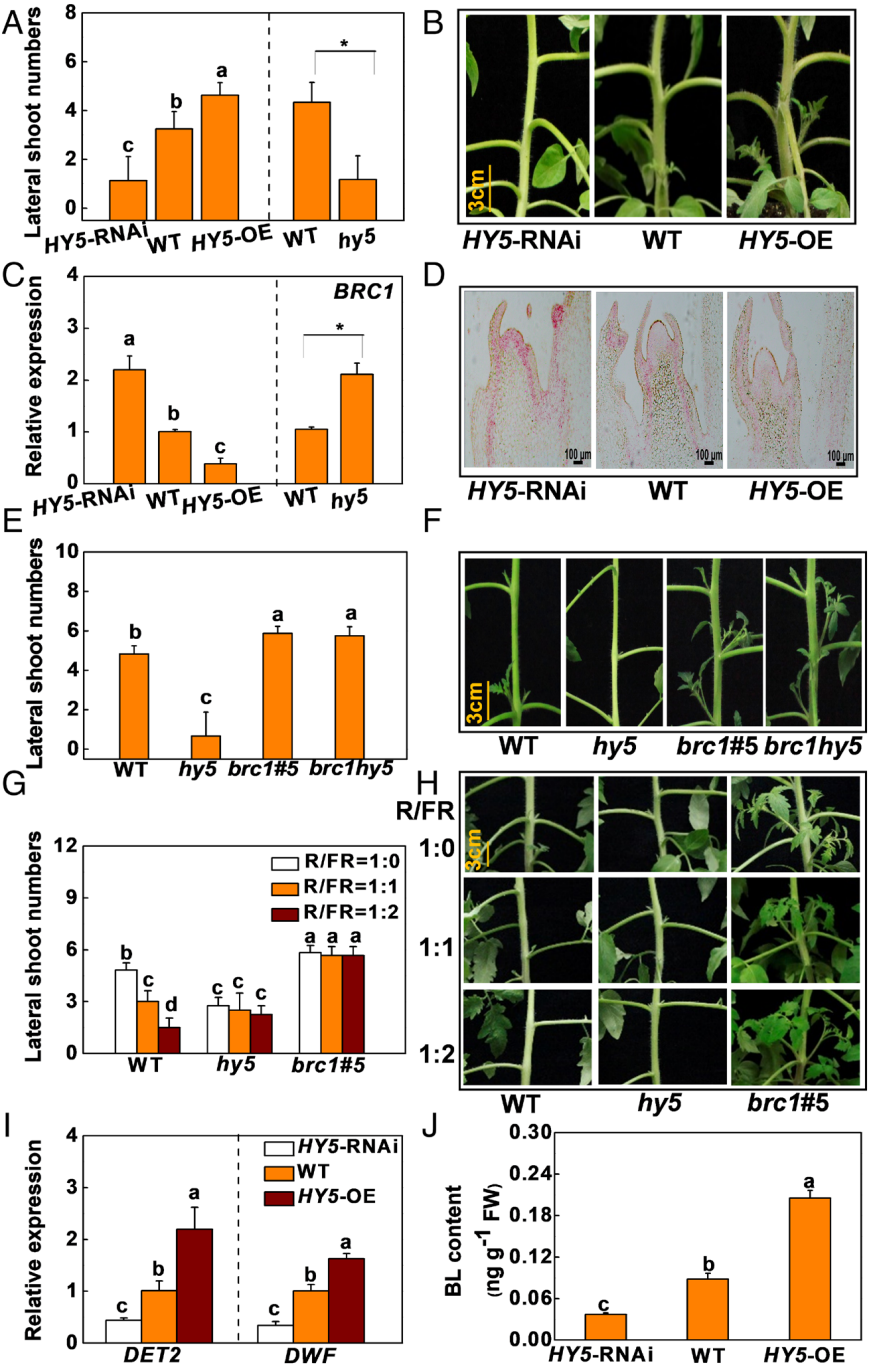
HY5 promotes bud outgrowth by functioning upstream of *BRC1* and BR in the light control of bud outgrowth in tomato. (*A*–*C*) The lateral shoot numbers, bud outgrowth phenotypes, and transcript level of *BRC1* in the lateral buds of *HY5*-RNAi, WT, and *HY5*-overexpressing (*HY5-*OE) plants or in WT plant and *hy5* mutant. (*D*) In situ hybridization of mRNA of *BRC1* in the lateral buds. (*E* and *F*) The lateral shoot numbers and bud outgrowth phenotypes of WT plant, *hy5*, *brc1* (*brc1* #5), and *brc1hy5* mutants. (*G* and *H*) The lateral shoot numbers and bud outgrowth phenotypes of WT plant, *hy5*, and *brc1* (*brc1* #5) mutants in response to changes in red-to-far-red ratio (R/FR). (*I*) Transcript of *DET2* and *DWF* in the lateral buds of *HY5*-RNAi, WT, and *HY5*-OE plants. (*J*) BL level in the lateral buds of *HY5*-RNAi, WT, and *HY5*-OE plants. For *A*–*F* and *I* and *J*, plants were grown under WL. All samples were taken at 12 AM. *ACTIN2* and *UBI3* were used as reference genes, and the gene expression in WT was defined as 1. Data are presented as the means of replicates ± SD. n = 12 to 15 in *A*, *E*, and *G* and n = 3 to 4 in *A*, *C*, *I* and *J*. Different letters or * indicate significant differences (*P *< 0.05) according to Tukey’s test or Student’s *t* test (*P* < 0.05), respectively.

To examine the role of HY5 in the light-regulated bud outgrowth, CRISPR/cas9 *hy5* mutants, *HY5*-RNAi, and *HY5*-overexpression (*HY5-*OE) lines were generated and used in the following experiments (*SI Appendix*, Fig. S4*A*). Both *HY5*-RNAi plants and *hy5* mutants had decreased number and total length of the lateral shoots with increased level of *BRC1* transcripts compared to the WT, while the *HY5-*OE plants exhibited increased number and total length of the lateral shoots with decreased level of *BRC1* transcripts (Fig. 2 *A*–*C* and *SI Appendix*, Fig. S4 *B* and *C*). In situ hybridization analysis revealed that the level of *BRC1* mRNAs in the meristems and leaf primordia of the axillary buds were higher in the *HY5*-RNAi plants than those of the WT, whereas the levels of *BRC1* transcripts were barely detectable in the *HY5-*OE buds (Fig. 2*D* and *SI Appendix*, Fig. S4*D*). Moreover, transcriptional suppression of *HY5* in the *CRY1a*-OE plants by RNA interference (*CRY1a*-OE*HY5-RNAi) (*SI Appendix*, Fig. S5 *A*–*D*) or virus-induced gene silencing (VIGS) of *HY5* (pTRV-*HY5*) (*SI Appendix*, Fig. S5 *E*–*I*) compromised *CRY1a*-OE-induced lateral bud outgrowth and increased the levels of *BRC1* transcripts. Taken together, these findings indicate that HY5 acts downstream of these photoreceptors to promote the growth of the lateral buds through inhibition of the expression of *BRC1* in the lateral buds in tomato.

To assess the role of BRC1 in HY5-regulated bud outgrowth, we silenced *BRC1* in the *HY5*-RNAi and WT plants (*SI Appendix*, Fig. S6*A*). Bud outgrowth was significantly increased in the *BRC1*-silenced plants under WL (*SI Appendix*, Fig. S6 *B*–*D*). We next generated *brc1* and *brc1hy5* mutants (*SI Appendix*, Fig. S7*A*). In sharp contrast to the *hy5* mutants, the *brc1* mutants (*brc1 #5, brc1 #9*) had increased number and total length of lateral shoots (Fig. 2 *E* and *F* and *SI Appendix*, Fig. S7 *B*–*D*). Crucially, the double *brc1hy5* mutation rescued the bud outgrowth phenotype in the *hy5* mutants. The *brc1hy5* mutants had similar number and total length of lateral shoots as the *brc1* mutants (Fig. 2 *E* and *F* and *SI Appendix*, Fig. S7*E*). This genetic evidence supports the hypothesis that *BRC1* acts downstream of HY5 to regulate bud outgrowth in tomato.

To further verify that HY5 and BRC1 play important roles in the light regulation of shoot branching, WT, *hy5,* and *brc1* #5 plants at the eight-leaf stage were preacclimated in the dark for 12 h and then were placed under different R/FR light treatment. Decreases in the R/FR light ratios led to significant decreases in number and total length of lateral shoots in the WT plants. However, changes in the R/FR light ratios had little effect on the number and total length of lateral shoots in the *hy5* or *brc1* mutants (Fig. 2 *G* and *H* and *SI Appendix*, Fig. S8). In addition, mutations in *HY5* abolished the R/FR light ratio-induced changes in the levels of *BRC1* transcripts (*SI Appendix*, Fig. S8). Furthermore, the levels of transcripts encoding the BR biosynthesis proteins *DET2* and *DWF* and the level of BL accumulation were decreased in the *HY5*-RNAi buds but increased in those of the *HY5*-OE plants (Fig. 2 *I* and *J*). These observations support the concept that HY5 promotes bud outgrowth by functioning upstream of BRC1 and BR in the light control of bud outgrowth in tomato.

### HY5 Functions as a Transcription Factor Regulating *BRC1*, *DET2*, and *DWF* Transcripts in Tomato.

**Fig. 3.**
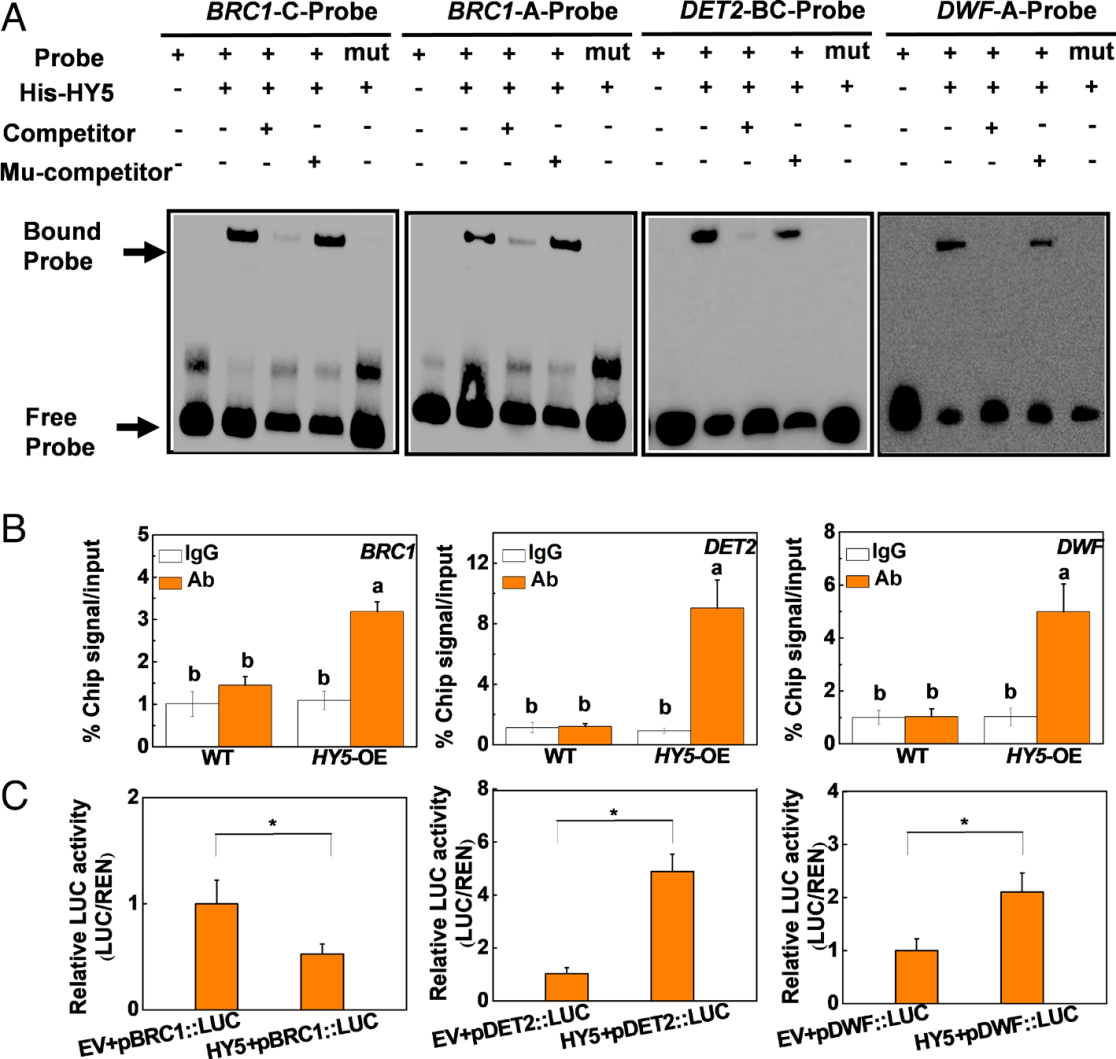
HY5 is involved in the light regulation of bud outgrowth through transcriptional regulation of *BRC1, DET2,* and *DWF* in tomato. (*A*) EMSAs to test the ability of HY5 to bind to the promoters of *BRC1, DET2,* and *DWF*. (*B*) ChIP-qPCR analysis of HY5-binding ability to the promoters of *BRC1, DET2,* and *DWF*. (*C*) Dual-luciferase assays for the regulatory effect of HY5 on the expression of *BRC1, DET2,* and *DWF*. The ratio of LUC/REN of the EV plus promoter was set as one. Data are presented as the means of three replicates ± SD. Different letters or * indicate significant differences (*P *< 0.05) according to Tukey’s test or Student’s *t* test (*P < 0.05), respectively.

We next examined whether HY5 functions downstream of light signals through the transcriptional regulation of *BRC1* and BR biosynthesis-related genes. Searching the promoters of the tomato *BRC1, DET2,* and *DWF* genes revealed several HY5 binding motifs, such as G-BOX (CACGTG), A-BOX (TACGTA), Z-BOX TACGTG, and C-BOX (GTCANN) motifs in these genes (*SI Appendix*, Fig. S9 *A*–*C*). Electrophoretic mobility shift assays (EMSAs) confirmed that HY5 can bind to *BRC1, DET2,* and *DWF* DNA probes containing A-box, G-box, and C-box element (Fig. 3*A* and *SI Appendix*, Fig. S9*D*). However, HY5 failed to bind to mutant probes, demonstrating the specificity of the binding of HY5 to these promoters. Moreover, ChIP-qPCR analysis provided further evidence that HY5 binds to the *BRC1, DET2,* and *DWF* promoters (Fig. 3*B*). In addition, dual-luciferase assays showed that HY5 transinhibits the promoter of *BRC1* but transactivates the promoters of *DET2* and *DWF* (Fig. 3*C* and *SI Appendix*, Fig. S9*E*). Collectively, these findings demonstrate that HY5 suppresses *BRC1* expression and promotes the expression of the *DET2* and *DWF* genes through binding to their promoters. Taken together, these results demonstrate that HY5 directly participates in the regulation of *BRC1* transcription and BR synthesis in tomato.

### BRC1 Regulates CK and GA Metabolism in Lateral Buds in Tomato.

**Fig. 4.**
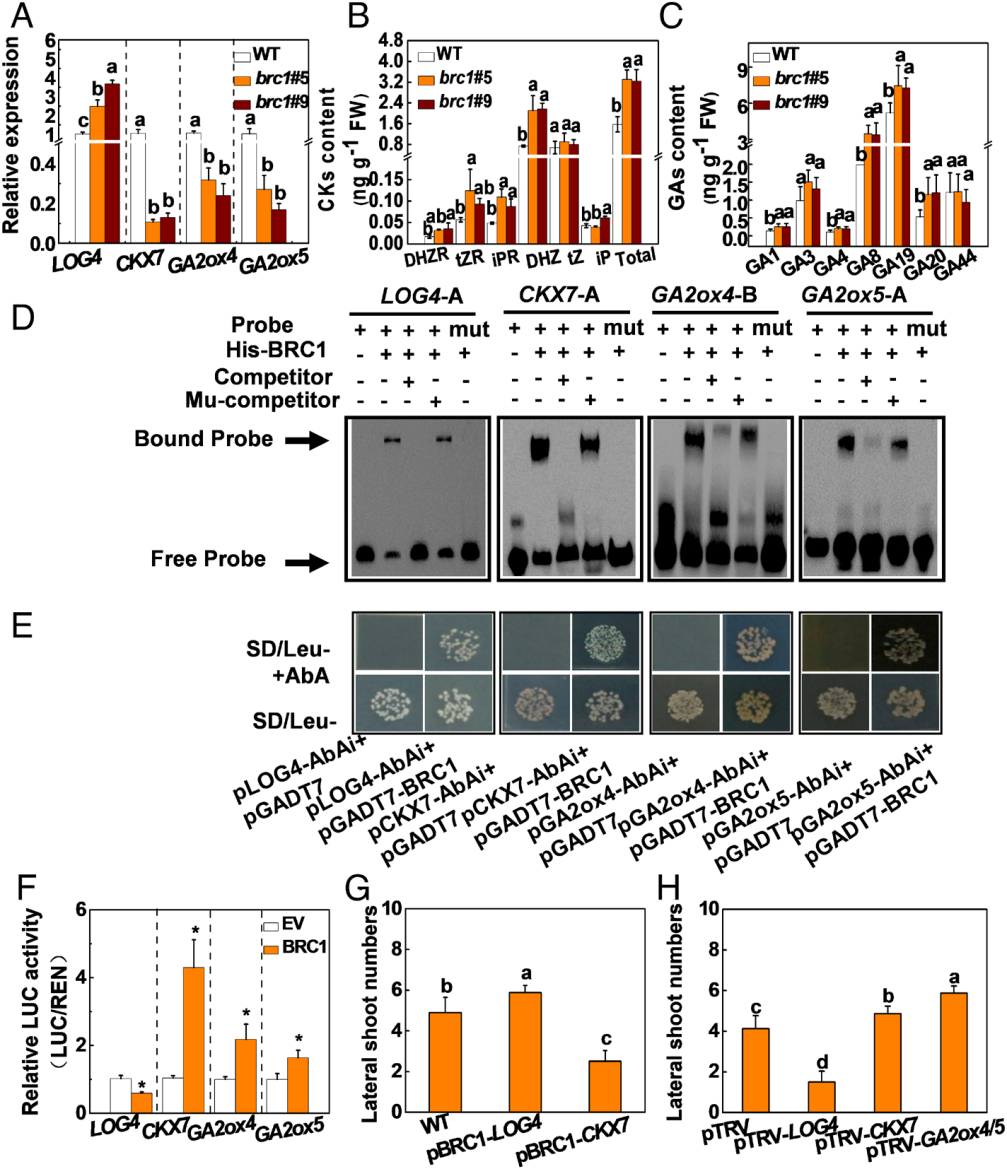
BRC1 is involved in CK and GA metabolism in the lateral buds in tomato. (*A*) Transcript of *LOG4*, *CKX7*, *GA2ox4,* and *GA2ox5* in the lateral buds of WT, *brc1* #5, and *brc1* #9 plants. *ACTIN2* and *UBI3* were used as reference genes, and the gene expression in WT was defined as 1. (*B* and *C*) CK and GA content in the buds of WT, *brc1* #5, and *brc1* #9 plants. (*D*) EMSAs to test the binding ability of BRC1 to the promoters of *LOG4*, *CKX7*, *GA2ox4,* and *GA2ox5*. The assays were repeated three times, and similar results were obtained. (*E*) Yeast one-hybrid analysis of BRC1 binding to the promoters of *LOG4*, *CKX7*, *GA2ox4,* and *GA2ox5*. (*F*) Dual-luciferase assays for the regulatory effect of BRC1 on the expression of *LOG4*, *CKX7*, *GA2ox4,* and *GA2ox5*. The ratio of LUC/REN of the EV plus promoter was set as 1. (*G*) Elevated expression of *LOG4* or *CKX7* driven by the *BRC1* promoter (pBRC1–*LOG4* or pBRC1–*CKX7*) altered bud outgrowth. (*H*) Silencing *LOG4 and CKX7* and cosilencing *GA2ox4* and *GA2ox5* altered bud outgrowth. All plants were grown under WL. Samples were taken at 12 AM. Data are presented as the means of replicates ± SD. n = 3 to 4 in *A*–*C* and *F* and n = 12 to 15 in *G* and *H*. Different letters indicate significant differences (*P *< 0.05) according to Tukey’s test (*P* < 0.05).

To identify the downstream targets of *BRC1* and the associated regulatory network, RNA-Seq analysis was performed on the lateral buds from the 4th stem node of WT, *brc1* #5, and *brc1* #9 plants at the eight-leaf stage under WL at 11 AM. Using a false discovery rate (FDR) of <0.05 and a fold change >1 as the significance cutoffs, 3,958 differentially expressed genes (DEGs) were identified in both lines of *brc1* #5 and *brc1* #9 compared to WT. Of these DEGs, 2,301 transcripts were significantly increased and 1,655 transcripts were decreased in the buds of the *brc1* #5 and *brc1* #9 mutants compared to those of the WT plants (*SI Appendix*, Fig. S10*A* and Dataset S1). The subsets of genes encoding proteins involved in CK and GA biosynthesis and catabolism were enriched, while other genes encoding proteins involved in auxin and ABA signaling were not significantly changed (Dataset S2). Heat map analysis indicated that the CK synthesis gene *LOG4*, the CK degradation gene *CKX7,* and the GA degradation genes *GA2ox4* and *GA2ox5* were differentially expressed in the WT, *brc1* #5, and *brc1* #9 plants (*SI Appendix*, Fig. S10 *B* and *C*). qRT-PCR confirmed that the levels of *LOG4* transcripts were increased by between 147% and 266%, while those of *CKX7* decreased by between 88% and 90% in the two *brc1* mutants relative to the WT plants (Fig. 4*A*). Meanwhile, the levels of *GA2ox4* and *GA2ox5* transcripts were significantly decreased in the lateral buds of *brc1* #5 and *brc1* #9 plants, compared to the WT plants. UPLC/MS/MS analysis revealed that the levels of total CKs (iP, iPR, tZ and tZR, DHZ, and DHZR) were significantly increased in the lateral buds of *b rc1* #5 and *brc1* #9 plants compared to the WT (Fig. 4*B*). Crucially, the levels of the bioactive CK tZ and also DHZR, tZR, iPR, and DHZ were significantly higher in the *brc1* #5 and *brc1* #9 buds than those of the WT. In addition, the levels of the active GAs GA1, GA3, GA4 as well as their precursors GA8, GA19, and GA20 were significantly higher in the *brc1* #5 and *brc1* #9 buds than those in the WT, with increases between 33% and 126% (Fig. 4*C*). However, the indole-3-acetic acid (IAA) levels in the lateral buds of the *brc1* #5 and *brc1* #9 mutants were only slightly lower or not significantly different from those of the WT (*SI Appendix*, Fig. S11*A*). There was also no significant difference in the ABA levels of the lateral buds between the WT and *brc1* #5 or *brc1* #9 plants (*SI Appendix*, Fig. S11*B*). Meanwhile, no significant differences were observed in the number and total length of lateral shoots between the WT and ABA synthesis mutant *not* (*SI Appendix*, Fig. S11 *C*–*E*). Taken together, these observations suggest that increased accumulation of CK and GA may contribute to the increased lateral outgrowth in the *brc1* mutants in tomato.

### BRC1 Directly Binds to the *LOG4, CKX7, GA2ox4,* and *GA2ox5* Promoters In Vitro and In Vivo.

To further dissect the regulatory role of BRC1 in the CK and GA signaling pathways, cis-acting elements in the genome sequences of *LOG4*, *CKX7*, *GA2ox4,* and *GA2ox5* genes were analyzed. Several putative elements in the promoters of *LOG4, GA2ox4, GA2ox5,* and *CKX7* were identified (*SI Appendix*, Fig. S12*A*). The EMSA revealed that BRC1 was bound to the DNA probes for *LOG4*-A (TGGGCC motif), *CKX7*-A (GGGCCCAA motif), *GA2ox4-B* (GGGACCAT motif), and *GA2ox5-A* (GGTGCCCT motif). Moreover, the binding was successfully outcompeted by unlabeled DNA probes, but not by unlabeled mutant probes in which the TGGGCC, GGGCCCAA, GGGACCAT, GGTGCCCT motifs were replaced by the TAAAAA, AAACAAAA, AAAAAAAT, AATGAACT motifs, respectively. In addition, BRC1 failed to bind to mutant probes, demonstrating the specificity of BRC1 binding to the *LOG4*, *CKX7*, *GA2ox4*, and *GA2ox5* promoters (Fig. 4*D*). Yeast one-hybrid assay demonstrated that yeast cells containing the *P_LOG4_*−, *P_ckx7_*−, *P_GA2ox4_*−, and *P_GA2ox5_*− bait vectors and the BRC1-AD vector were able to grow on SD/Leu- media containing aureobasidin A (AbA). In contrast, transformants without BRC1 failed to grow on this media (Fig. 4*E*). Dual-luciferase assays showed that the activity of the *LOG4* promoter was inhibited by BRC1 binding, whereas the activities of the *CKX7*, *GA2ox4*, and *GA2ox5* promoters were activated by BRC1 binding (Fig. 4*F* and *SI Appendix*, Fig. S12*B*). Taken together, these in vitro and in vivo experiments demonstrate that BRC1 represses the expression of *LOG4* but activates the expression of *CKX7*, *GA2ox4*, and *GA2ox5* by directly binding to their promoters in tomato.

### The Local Transcriptional Regulation of *LOG4, CKX7, GA2ox4*, and *GA2ox5* Is Required for Bud Outgrowth in Tomato.

Based on the results presented above, it is reasonable to speculate that the *BRC1*-mediated inhibition of branch development is dependent on its role in the regulation of *LOG4*, *CKX7*, *GA2ox4*, and *GA2ox5* expression. To test this hypothesis, we expressed *LOG4*, *CKX7*, *GA2ox4*, and *GA2ox5* under the control of the *BRC1* promoter (pBRC) in WT plants. Two representative pBRC1–LOG4 and pBRC1–CKX7 transgenic lines (*SI Appendix*, Fig. 13 *A*–*C*) were obtained. Unfortunately, the callus of the pBRC1–*GA2ox4* and pBRC1–*GA2ox5* transformations failed to grow, possibly because of the reduced accumulation of GAs. The specific accumulation of the LOG4 protein in the pBRC1–LOG4 line significantly promoted bud outgrowth (Fig. 4*G* and *SI Appendix*, Fig. S14 *A* and *B*). Conversely, the specific accumulation of the CKX7 protein in the pBRC1–CKX7 line significantly decreased the number and total length of lateral shoots. We also silenced *LOG4* and *CKX7* separately and cosilenced the *GA2ox4* and *GA2ox5* genes using VIGS. The resultant lines had a reduction in the levels of these transcripts by 68 to 85% (*SI Appendix*, Fig. S14*C*). In consistence to the above results, we found that the silencing of *LOG4* resulted in decreased number and total length of lateral shoots. In contrast, silencing of *CKX7* or cosilencing of *GA2ox4* and *GA2ox5* led to increases in the number and total length of lateral shoots compared to the corresponding empty vector (EV) the plasmid of Tobacco Rattle Virus (pTRV) controls (Fig. 4*H* and *SI Appendix*, Fig. S14 *D* and *E*). Taken together, these results show that BRC1 affects the CK and GA signaling pathways through the transcriptional regulation of the *LOG4*, *CKX7*, *GA2ox4*, and *GA2ox5* genes, thereby suppressing bud outgrowth in tomato.

### Temporal–Spatial Regulation of BRC1 Transcript Accumulation Is Linked to Diurnal Changes in Bud Outgrowth in Tomato.

**Fig. 5.**
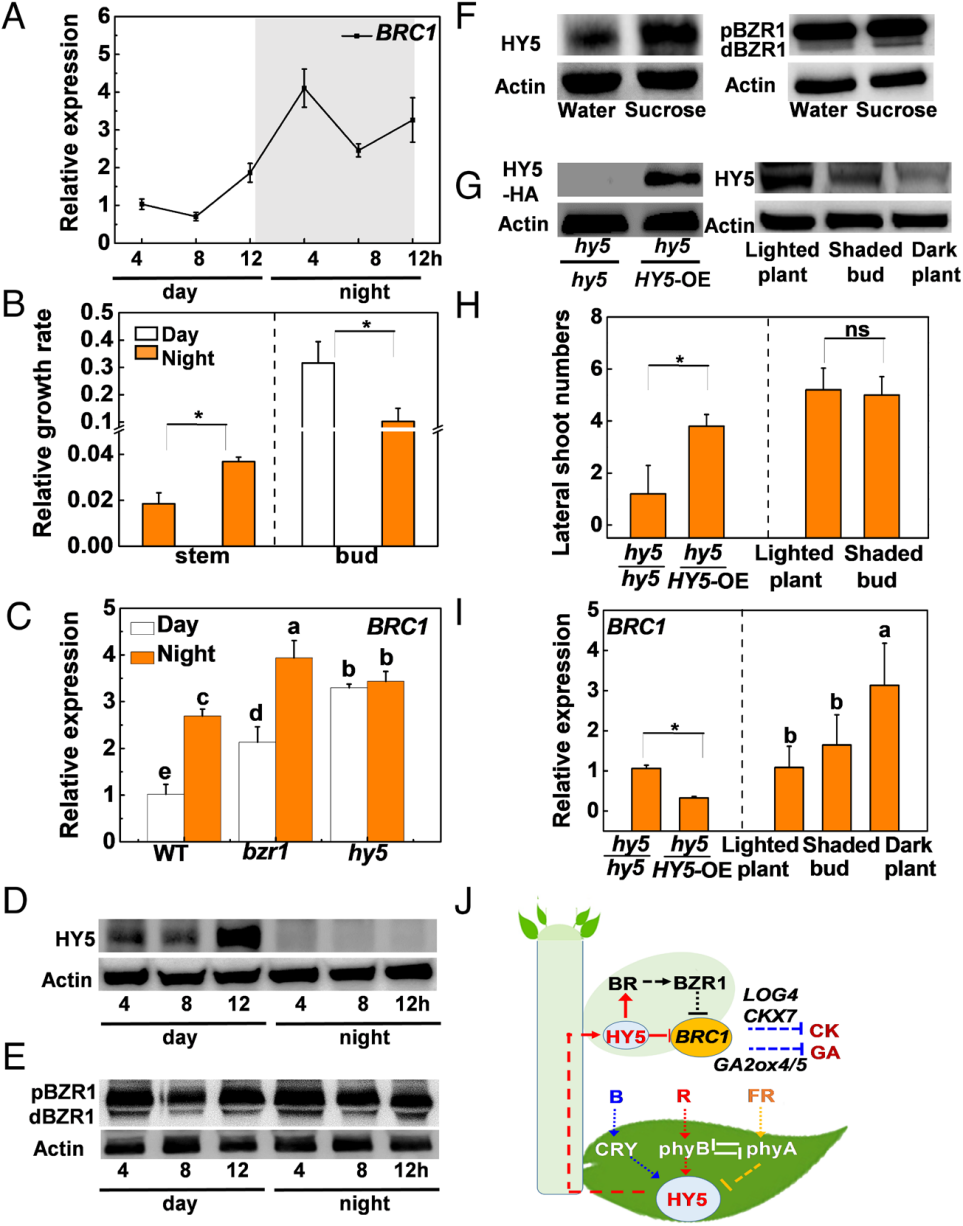
Temporal and spatial mobility characteristics of HY5 contribute to the diurnal bud outgrowth in tomato. (*A*) Diurnal changes in the transcript level of *BRC1* in the lateral buds. (*B*) The relative growth rate of lateral buds and stem in the day and in the night. The relative lateral bud growth rate is calculated as (L1 to L0)/L0, where L0 is the bud length at 8 AM or 8 PM and L1 is length after exposure to light or dark for 12 h. (*C*) The transcript level of *BRC1* in the lateral buds of WT plant, *bzr1,* and *hy5* mutants. (*D* and *E*) Diurnal changes in the accumulation of HY5 and BZR1 proteins in the lateral buds. (*F*) Accumulation of HY5 and BZR1 in lateral buds in response to sucrose application. (*G*–*I*) HY5 protein accumulation, bud outgrowth, and expression of *BRC1* for the *hy5* lateral buds in the grafts or in the bud shading treatment. All plants were grown under WL except the dark plant treatment. *ACTIN2* and *UBI3* were used as reference genes. Actin was used as a loading control for the western blot analysis. Samples were taken at 12 AM and 12 PM in *C* and 12 AM in *F*, *G*, and *I*. Data are presented as the means of replicates ± SD. n = 12 to 15 in *H* and n = 3 to 4 in *A*–*C* and *I*. Different letters or * indicate significant differences (*P *< 0.05) according to Tukey’s test or Student’s *t* test (*P < 0.05), respectively. (*J*) The model in which HY5 integrates light signaling and BRC1 to regulate bud outgrowth in tomato. Arrows indicate activation and blunt-ended lines indicate inhibition.

To obtain further insights into the mechanism that control light-regulated bud outgrowth, we examined the diurnal changes in *BRC1* transcript levels, the accumulations of the HY5 and BZR1 proteins, and bud growth rates. The lateral buds had lower levels of *BRC1* transcripts in the day than at night (Fig. 5*A*). In contrast to stem elongation, the lateral buds had higher relative growth rates during the day than at night (Fig. 5*B*). qRT-PCR analysis showed that the *hy5* mutants showed higher daytime levels of *BRC1* transcripts than those of *bzr1* mutant and WT, while the WT plants had the lowest levels of *BRC1* transcripts during the day. The WT, *hy5* mutants, and *bzr1* mutants all had higher levels of *BRC1* at night. The WT plants had the lowest levels of *BRC1* transcripts at night, but no significant difference in the accumulation of *BRC1* transcripts was observed in the *hy5* mutants during the day or at night (Fig. 5*C*). Western blotting and qRT-PCR analysis revealed that lateral buds accumulated greater amounts of the HY5 protein and higher levels of *HY5* transcripts during the day than at night (Fig. 5*D* and *SI Appendix*, Fig. S15*A*). However, no substantial differences in BZR1 protein accumulation or in *BZR1* transcript levels were observed in the lateral buds sampled in the day or at night (Fig. 5*E* and *SI Appendix*, Fig. S15*B*). Meanwhile, the buds accumulated more sucrose, the transport sugar in tomato phloem, during the day than at night. This diurnal effect on sucrose accumulation was greatest in the *HY5*-OE plants and least pronounced in the *HY5*-RNAi plants compared to the WT plants (*SI Appendix*, Fig. S16*A*). The foliar application of sucrose significantly promoted bud outgrowth and increased the accumulation of both HY5 and dBZR1 proteins but decreased *BRC1* transcript levels in the buds (Fig. 5*F* and *SI Appendix*, Fig. S16 *B*–*E*). Taken together, these data show that HY5 contributes to the diurnal changes in *BRC1* transcript accumulation and bud growth rate in tomato.

To determine the contribution of the HY5 pool in the leaves to the regulation of bud outgrowth, we grafted the shoots of *hy5* plants (with four true leaves) onto *HY5*-OE plants with four leaves (*hy5/HY5*-OE), that had been transformed to express an HY5 with the C-terminal fused with the hemagglutinin (HA) tag, or *hy5* plants with four leaves (*hy5/hy5*), respectively. The HY5–HA tag fusion protein was detectable in the scion lateral buds (*hy5*) of the hy5/*HY5*-OE grafts but not in those of *hy5/hy5* grafts (Fig. 5*G*). In addition, increased levels of *HY5* transcripts were detected in the *hy5* scion lateral buds of the *hy5/HY5*-OE line (*SI Appendix*, Fig. S17*A*). Crucially, the number and total length of lateral shoots were significantly increased in the *hy5* scion in the *hy5 /HY5*-OE grafts. This was associated with a decreased accumulation of *BRC1* transcripts relative to the self-grafted *hy5/hy5* combination (Fig. 5 *H* and *I* and *SI Appendix*, Fig. S17 *B* and *C*). In support of the role of HY5 from the leaves in the bud outgrowth, shading the lateral buds with tin foil did not evidently alter the HY5 accumulation and *BRC1* transcript in the shaded buds and had little effects on the bud outgrowth (Fig. 5 *G*–*I* and *SI Appendix*, Fig. S17 *D* and *E*). In comparison, buds in shaded plants showed a higher transcript of *BRC1* and a low accumulation of HY5 than those of shaded buds and buds in the plants under light. These results together suggest that HY5 acts as a systemic signal that can move from the leaves to the lateral buds in the regulation of the growth of lateral buds in tomato.

## Discussion

The signaling pathway that controls light-regulated branching has remained elusive. Here, we provide convincing evidence supporting the concept that HY5 is crucial to the control of shoot branching through suppression of *BRC1* transcription via both direct action and BZR1-mediated indirect action in tomato. Furthermore, the results presented here demonstrate that BRC1 suppresses bud outgrowth by decreasing CK and GA accumulation through the transcriptional regulation of the *LOG4*, *CKX7*, *GA2ox4*, and *GA2ox5* genes. Crucially, we present evidence showing that the light-activated movement of HY5 produced in the leaves regulates bud outgrowth, and that HY5, as a mobile systemic signal, contributes to the diurnal regulation of bud outgrowth in tomato. Based on these findings, we propose a model, in which HY5 integrates light signaling and BRC1 functions to regulate bud outgrowth in tomato (Fig. 5*J*).

### Light Regulates Bud Outgrowth via BR-Dependent and -Independent Pathways in Tomato.

Light quality is an important environmental cue that greatly influences plant architecture, particularly in shade avoidance responses. The low R/FR light ratio-mediated suppression of bud outgrowth is commonly thought to be caused by an increase in polar auxin transport and dependent on SL signaling. Decreased CK accumulation might also be important in this context, because CK may promote bud outgrowth by antagonizing auxin and SL (12, 19, 21, 24). Our recent studies show that BR mediates the action of these phytohormones via the type-B response regulator (RR10)-dependent regulation of BR synthesis, which leads to the suppression of *BRC1* expression by BR-activated BZR1 (20). Consistent with these findings, we show here that the low R/FR ratio-induced suppression of bud outgrowth was accompanied with decreases in the levels of *DET2* and *DWF* transcripts and the accumulation of BL and BZR1 protein, followed by an increase in *BRC1* transcript in the buds (Fig. 1 *A*–*C* and *SI Appendix*, Fig. S1). However, BR signaling alone is not sufficient to mediate the light-regulated bud outgrowth because mutations in BR synthesis gene (*dwf*) and in key signaling component (*bzr1*) did not abolish the R/FR light ratio-induced changes in bud outgrowth and *BRC1* transcript abundance (Fig. 1 *D* and *E* and *SI Appendix*, Fig. S2). These findings show that other BZR1-independent signaling pathways are involved in light-dependent regulation of bud outgrowth in tomato.

We present data showing that photoreceptors are directly involved in the regulation of bud outgrowth. As observed in earlier studies (12, 24), decreasing the R/FR light ratio in the growth environment or defects in *PHYB1B2* inhibit bud outgrowth and increase the levels of *BRC1* transcripts in the lateral buds. Moreover, defects in *PHYA* promote bud outgrowth, while defects in *PHYB1B2* or *CRY1a* suppress bud outgrowth (Fig. 1 *F* and *G* and *SI Appendix*, Fig. S3). These findings suggest that *PHYB1B2* and also *PHYA* and *CRY1a* are involved in the regulation of bud outgrowth in tomato. Furthermore, our results demonstrate that the transcriptional regulation of *BRC1* is required for light regulation of bud outgrowth because defects in *BRC1* abolished R/FR light ratio-induced changes in bud outgrowth (Fig. 2 *G* and *H* and *SI Appendix*, Fig. S8*A*). These findings suggest that BRC1 mediates photoreceptor-regulated bud outgrowth in tomato.

### HY5 Is Essential for the Release of Apical Dominance by Acting as the Transcription Factor Regulating the Expression of *BRC1*, *DET2*, and *DWF* in Tomato.

HY5 acts downstream of multiple photoreceptors to regulate many physiological and biological processes in the light-dependent control of plant growth and development (32, 33, 35). In agreement with early studies in the tomato leaves (36–39), HY5 accumulation in the buds is also dependent on light quality and photoreceptor (Fig. 1 *H* and *I*). Importantly, the results presented here provide several levels of evidence showing that HY5 is directly involved in the light-dependent regulation of bud outgrowth. First, *HY5*-RNAi and *hy5* mutants showed reduced bud outgrowth and increased *BRC1* transcript levels. In contrast, overexpression of *HY5* promoted lateral bud outgrowth, together with suppression of *BRC1* expression (Fig. 2 *A*–*D* and *SI Appendix*, Fig. S4). Second, the accumulation of *HY5* transcripts and protein was decreased under low R/FR conditions, and also in *phyB1B2* and *cry1a* mutants. In contrast, the levels of *HY5* transcripts and protein were increased in *CRY1a*-OE and *phyA* plants, which is in good agreement with the branching phenotypes (Fig. 1 *F*, *H*, and *I* and *SI Appendix*, Figs. S1 and S3). In addition, decreasing the R/FR light ratio had little effect on bud outgrowth in the *hy5* mutants (Fig. 2 *G* and *H* and *SI Appendix*, Fig. S8*A*). Third, suppression of *HY5* transcript in the *CRY1a*-OE lines resulted in decreased number and total length of lateral shoots and increased *BRC1* transcript levels (*SI Appendix*, Fig. S5). Crucially, a series of in vitro and in vivo experiments including EMSA, ChIP-qPCR, and dual-luciferase assays provide evidence that HY5 acts as a transcriptional regulator of the expression of *BRC1* (Fig. 3 and *SI Appendix*, Fig. S9). These results demonstrate that HY5 plays important roles not only in photomorphogenesis (33, 34), but also in the light-dependent regulation of plant architecture in tomato by integrating information from different light signals.

Previously, we reported that auxin, CK, SL, and sugar alter BR synthesis and bud outgrowth through the action of RR10, a transcriptional factor that is involved in CK signaling (20). Here, we provide evidence showing that HY5 positively regulates BR synthesis through the transcriptional activation of the BR biosynthesis genes *DET2* and *DWF* (Figs. 2 *I* and *J* and 3 and *SI Appendix*, Fig. S9). These results therefore suggest that both RR10-dependent and HY5-dependent pathways are involved in the control of BR biosynthesis. Since both HY5 and the BR signaling component BZR1 function as suppressors of *BRC1* expression, the HY5-mediated promotion of bud outgrowth is partially achieved through BR/BZR1-mediated suppression of *BRC1* in response to changes in light quality.

### BRC1 Inhibits Bud Outgrowth by Suppressing the Accumulation of CK and GA in Tomato.

The BRC1 transcription factor is a central integrator of signals that control bud outgrowth, while to date, relatively few target genes for BRC1 such as those involved in polar auxin transport and ABA signaling have been identified (1, 31). Similarly, information concerning the role of BRC1 in CK signaling and GA signaling was also lacking, although these phytohormones play crucial roles in cell division, cell enlargement, sprouting, seed germination, and flowering (40). In this study, we found that the lateral buds of the *brc1* mutants accumulated higher levels of CKs and GAs than those of the WT, but there were no significant differences in the accumulation of IAA or ABA between the WT and *brc1* mutants (Fig. 4 *B* and *C* and *SI Appendix*, Fig. S11 *A* and *B*). Consistent with this, RNA-Seq analysis showed that DEGs involved in CK and GA metabolism were enriched, but potential targets involved in auxin and ABA signaling were unchanged (*SI Appendix*, Fig. S10 and Dataset S2). In vitro and in vivo experiments, together with qRT-PCR, revealed that BRC1 represses the expression of the CK synthesis gene *LOG4*, but activates the expression of the CK degradation gene *CKX7* and the GA degradation pathway genes *GA2ox4* and *GA2ox5* by directly binding to the promoters of these genes (Fig. 4 *A* and *D*–*F*). In addition, genetic evidence indicated that *LOG4*, *CKX7*, *GA2ox4*, and *GA2ox5* act downstream of BRC1. Elevated expression of *LOG4* driven by the *BRC1* promoter resulted in excessive shoot branching, while elevated expression of *CKX7* driven by the BRC1 promoter resulted in less shoot branching (Fig. 4*G* and *SI Appendix*, Figs. S13 and S14 *A* and *B*). Similarly, silencing of *LOG4* resulted in less shoot branching, while silencing of *CKX7*, *GA2ox4,* and *GA2ox5* resulted in excessive shoot branching (Fig. 4*H* and *SI Appendix*, Fig. S14 *C*–*E*). Taken together, these results demonstrate that BRC1 is a key repressor of shoot branching by directly inhibiting the activity of CK and GA in tomato.

### Temporal and Spatial Mobility Characteristics of HY5 Contribute to the Diurnal Regulation of Bud Outgrowth in Tomato.

Light and light-induced changes in HY5 accumulation are known to inhibit hypocotyl elongation in photomorphogenesis (33, 34). To our surprise, bud outgrowth occurred mostly in the day (Fig. 5*B*). This agrees with the diurnal changes in the levels of *BRC1* and HY5 accumulation in the buds (Fig. 5 *A* and *D*), but contrasts with light-regulated diurnal changes in stem elongation in our study and hypocotyl elongation in other studies (41). Apparently, the HY5-mediated suppression of *BRC1* expression in lateral buds could explain the observed differences in plant growth regulation. The time course of *BRC1* transcript accumulation reveals that HY5 suppresses *BRC1* expression during the day, while BZR1 inhibits the expression of *BRC1* throughout the day and night. BZR1 undergoes degradation in light in photomorphogenesis (42). However, only small diurnal changes in the accumulation of BZR1 were observed in the lateral buds of tomato plants (Fig. 5*E*). This finding is attributable to the higher accumulation of sucrose in the day. Sucrose may increase the stability of HY5 and BZR1 (20). Moreover, HY5 promotes sucrose accumulation by activating several starch degradation-related genes (39). To date, the factors that are required for the control of bud outgrowth following light perception have not been identified (43–45). In a previous study, sugar (but not auxin) was found to be the initial regulator of apical dominance (30). In this study, sugar promotion of bud outgrowth was associated with an increased accumulation of HY5 in the buds, suggesting that HY5 mediates, at least in part, the sugar-regulated bud outgrowth (*SI Appendix*, Fig. S16). Crucially, we found that HY5 plays a role in the regulation of bud outgrowth through movement from the leaves to the lateral buds. This concept is substantiated by the improved bud outgrowth and decreased abundance of *BRC1* transcripts in the *hy5* shoots grafted onto *HY5-*OE shoot (Fig. 5 *G*–*I* and *SI Appendix*, Fig S17). In agreement with this, shading the buds did not alter bud outgrowth with little or mild changes in the HY5 accumulation and *BRC1* expression in the buds (Fig. 5 *H* and *I*). Together, these findings suggest that HY5 functions as a universal systemic signal that is not only involved in the systemic regulation of nutrient utilization in the roots and photoprotection in the shoots (46, 47), but also in the regulation of plant architecture in tomato.

Prior to this study, the general consensus of opinion was that light regulates the central integrator BRC1 to control bud outgrowth by integrating hormone and sugar signals (48). The data presented here demonstrate that HY5-dependent light signaling alone is sufficient to regulate bud outgrowth in tomato. In addition, our findings reveal an important and previously unrecognized role for BRC1 in regulating CK and GA signaling. Moreover, these studies demonstrate that plants, with the aid of HY5, use different strategies to control stem elongation and bud outgrowth in order to adapt to the changes in light conditions. While the light-induced regulation in HY5 impairs stem elongation by suppressing GA signaling in photomorphogenesis, this process induces the activation of branches through BRC1-dependent regulation of CK and GA signaling in tomato (32). Such a spatial heterogeneity is likely to confer a physiological advantage and contributes to the capacity of plants to adjust light utilization and resource allocation in the shade avoidance response.

## Materials and Methods

### Plant Materials.

Tomato (*Solanum lycopersicum*) cv. Moneymaker (WT); cv. Condine Red (WT); cv. Ailsa Craig (WT); and the *cry1a, phyA*, *phyB1B2*, *not,* and *dwf* mutants were obtained from the Tomato Genetics Resource Center at the University of California, Davis (http://tgrc.ucdavis.edu). *CRY1a*-OE, *HY5-*OE, and *BZR1*-OE transgenic plants; *HY5-*RNAi plants; and *bzr1* mutant were generated as described previously (49–55). The CRISPR/Cas9 mutants of *hy5* and *brc1* (*brc1* #5, *brc1* #9 lines) in the cv. Condine Red background were generated similar to previous studies (20, 36). The mutation sites are shown in *SI Appendix*, Figs. S4*A* and S7*A*. The double-mutant *brc1hy5* was generated by crossing *brc1 #5* mutant and *hy5* mutant, and the homozygous F3 generation was used. *CRY1a*-OE*HY5-RNAi was generated by crossing *CRY1a*-OE and *HY5*-RNAi.

### Growth Condition and Light Treatment.

Tomato seeds were germinated in a growth medium composed of a mixture of peat, perlite, and vermiculite (3:1:1, v/v) in trays in a growth chamber. When the first true leaf was fully expanded, the seedlings were transplanted into plastic pots (10 cm diameter and 9 cm depth, one seedling per pot) containing the same medium and were watered every 3 d with Hoagland’s nutrient solution. The growth conditions were as follows: a 12-h photoperiod (8 AM to 8 PM) and temperature of 25/20 °C (day/night) under white light with photosynthetic photon flux density maintained at 400 µmol m^−2^ s^−1^. Light was supplied with LED (PHILIPS, Netherlands) with red (R): blue (B): far-red (FR) light ratio at 1:0.4:0.8 unless otherwise stated. At 8-10-leaf stage, buds longer than 4 to 5 mm were counted and the length of lateral buds was measured using a numeric caliper. For R/FR treatment, plants at the eight-leaf stage were preacclimated in the dark for 12 h and were then transferred to the different ratios of R/FR light (set as 1:0,1:1, and 2:1), for 7 d. LED lamps with peak wavelength at 735 nm (FR) and 660 nm (R) were used (PHILIPS, Netherlands).

### Grafting, Sucrose, and Bud Shading Treatments.

The *HY5*-RNAi and *HY5*-OE plants at the eight-leaf stage were used in this experiment. The leaves of the *HY5*-RNAi and *HY5*-OE plants at the eight-leaf stage were marked from no. 1 to 8 acropetally. The *HY5*-RNAi plants were cut between the 4th and the 5th leaf and the shoots with the 5th to 8th leaves were grafted onto another *HY5*-RNAi or *HY5*-OE plants with 1th to 4th leaves as described previously (47), which resulted in two combinations: *hy5/hy5* (*HY5-RNAi/HY5*-RNAi) and *hy5*/*HY5*-OE (*HY5*-RNAi/*HY5*-OE). After adaptation under dark with relative humidity of 90 to 100% for 4 d, the grafted plants were gradually exposed to white light up to a photosynthetic photo flux density of 400 μmol m^−2^ s^−1^ at temperature of 25 °C/20 °C for 6 d. After that, the grafted plants are ready for the subsequent experiment.

To determine the role of HY5 from the leaves in the bud outgrowth, buds together with stems of the WT plant at eight-leaf stage were shaded with tin foil while leaves were exposed to WL for 7 d before the determination of bud outgrowth and biochemical analysis. For the comparison, some plants were exposed to WL or covered with tin foil. For sugar feeding, each plant was sprayed with 15 mL sucrose solution at a concentration of 20 mM at 8 AM every day. The treatment lasted for 3 d.

### Cloning Procedures and Plant Transformation.

To generate the pBRC1–*LOG4* and pBRC1–*CKX7* transgenic plants, the full-length coding DNA sequence (CDS) of *SlLOG4* and *SlCKX7* excluding stop codons was obtained by PCR using specific primers shown in *SI Appendix*, Table S1. The PCR products were inserted behind the *BRC1* promoter in the binary plasmid vector pFGC1008-3HA (in which the CaMV 35S promoter was replaced with BRC1 promoter). The resulting pBRC1–*LOG4*–3HA and pBRC1–*CKX7*–3HA plasmids were introduced into *Agrobacterium tumefaciens* strain GV3101 by electroporation, after which the plasmids were introduced into *S. lycopersicum* cv. Condine Red plants. Transgenic plants were identified by western blot using an anti-HA monoclonal antibody (Abcam, ab18181). Two independent homozygous lines of the F2 progeny of pBRC1–*LOG4* and pBRC1–*CKX7* transgenic plants were used in this study.

### VIGS and *Agrobacterium*-Mediated Viral Infiltration.

For gene silencing, cDNA fragments of target genes were amplified using primers listed in *SI Appendix*, Table S2. The tobacco rattle virus (TRV) VIGS construct was used. Purified PCR products were cloned into pTRV2 vector using the ClonExpress II One Step Cloning Kit (C112, Vazyme, Nanjing, China). The plasmids were transformed into *A. tumefaciens* strain GV3101 after confirmation by sequencing. VIGS was performed as previously described (39). *A. tumefaciens* cultures carrying empty TRV1 and TRV2 vectors were also coinfiltrated as controls (pTRV). The infiltrated plants were grown at 23/21 °C (day/night) in a growth chamber with a 12 h day length for 30 d before the experiments (56, 57).

### Measurement of Phytohormones and Sucrose.

For the measurements, 0.1 g lateral buds in plants were sampled at 12 AM with the exception for the GA determination, in which 0.5 g samples were applied. For the determination of hormones, [26-^2^H_3_]brassinolide, [^2^H_6_]N^6^-isopentenyladenine (D-iP), [^2^H_6_]N^6^-isopentenyladenosineriboside (D-iPR), [^2^H_3_]dihydrozeatin (D-DHZ), [^2^H_3_]dihydro-zeatinriboside (D-DHZR), [^2^H_5_]*trans*-zeatin (D-tZ), and [^2^H_5_]*trans*-zeatinriboside (D-tZR) D_2_-GA_1_, D_2_-GA_4_, D_2_-GA_8_, D_2_-GA_9_, D_2_-GA_20_, and D_2_-GA_34_, D_6_-ABA, and D_6_-IAA (Olchemim, Olomouc, Czech Republic) were spiked into the extraction solutions as internal standards. All the measurements for hormones and sucrose were performed with ACQUITY UPLC^®^I-Class coupled to Waters Xevo^TM^ TQ-XS triple quadruple mass spectrometer as described previously (20, 39, 56, 58–60).

### Total RNA Extraction and qRT-PCR Analyses.

Total RNA was extracted from the lateral buds at the 4th stem node sampled at 12 AM unless otherwise described using an RNA extraction kit (TIANGEN, Beijing, China). Total RNA (1 µg) was reverse transcribed using the ReverTra Ace qPCR RT Kit (Toyobo, Osaka, Japan). qRT-PCR analyses were performed on a Light Cycler® 480 II Real-Time PCR Detection System (Roche, Basel, Switzerland). The PCR program showed predenaturation at 95 °C for 3 min, followed by 45 cycles of 95 °C for 30 s, 57 °C for 20 s, and 72 °C for 30 s. The gene-specific primers are shown in *SI Appendix*, Table S3. The relative expression levels were normalized to the expression level of the tomato housekeeping gene *ACTIN2* and *UBI3* (61).

### RNA Transcriptome Analyses Using RNA-Seq.

The lateral buds at the 4th stem node were collected from WT plants, *brc1* #5, and *brc1* #9 mutants grown under WL at 12 AM. Total RNA was isolated according to the RNeasy mini kit (QIAGEN), with an additional DNase I (QIAGEN) digestion step to remove any genomic DNA contamination. The cDNA library was prepared using the TruSeq RNA Sample Prep Kit (Illumina, San Diego, CA). Three biological replicates were conducted for RNA transcriptome analyses. The samples were clustered and sequenced on an Illumina Hiseq2500 (Berry Genomics Company). The cleaned reads were aligned to the tomato genome sequence SL4.00 (Sol Genomics Network) by the Hierarchical Indexing for Spliced Alignment of Transcripts (HISAT) software (version 0.1.6), allowing one mismatch to generate unique sequences. The transcript abundances were measured as fragments per kilobase of exon per million fragments mapped (FPKM) by Cufflinks 2.1.1. Cuffdiff 2 was then used to determine differential expression (FDR ≤ 0.05). Fold changes (log2 ratio) were calculated on the basis of FPKM. A log2 ratio > 1 or <−1 and FDR < 0.05 was considered to be the threshold for identifying (DEGs) (50).

### In Situ Hybridization.

Tomato lateral buds of WT, *HY5*-RNAi, and *HY5*-OE plants (about 2 mm) were fixed in 4% paraformaldehyde. Sample fixation, sectioning, and hybridization were conducted as described previously (20). In situ hybridization was performed according to the RNAscope 2.5 HD Detection Kit [Advanced Cell Diagnostics (ACD), California, USA] following the manufacturer’s protocol. *BRC1* probe and a negative control probe for an irrelevant bacterial gene dapB were provided by ACD. Slides were imaged on a Zeiss Axio Scope A1 microscope (Zeiss, Thueringen, Germany) with a Zeiss Axiocam 503 color camera and Zeiss ZEN imaging software.

### Protein Extraction and Western Blot.

Total protein was extracted from 0.05 to 0.1 g of lateral buds at the 4th stem node sampled at 12 AM unless otherwise described. Lateral buds were ground into powder in liquid nitrogen in 0.2 to 0.3 mL of extraction buffer (100 mM Tris-HCl, pH 8.0, 10 mM NaCl, 1 mM EDTA, 1% Triton X-100, 1 mM phenylmethylsulfonyl fluoride, and 0.2% β-mercaptoethanol). The extracts were mixed fully and centrifuged at 13000 g for 20 min at 4 °C, after which the extracted proteins were denatured at 95 °C for 10 min. For western blot, 100 μg denatured protein extracts were separated using 12% sodium dodecyl sulfate-polyacrylamide gel electrophoresis and then were transferred to nitrocellulose membranes (Millipore, Saint-Quentin, France). The HY5 protein was detected with a rabbit antibody against HY5 (Shanghai Jiayuan Bio Co., Shanghai, China). The HY5-HA, BZR1-HA, LOG4-HA, and CKX7-HA proteins were detected with commercial antibodies raised against anti-HA monoclonal antibody (Abcam, ab18181). Actin was used as a control for western-blot analysis. Each experiment was repeated three times.

### Recombinant Protein Purification and EMSA.

The pET-32a-His-HY5 vector was constructed as described previously (38, 58). The full-length *SlBRC1* CDS was amplified with the gene-specific primers listed in *SI Appendix*, Table S4. Purified PCR product was cloned into pET-32a vector using the ClonExpress II One Step Cloning Kit (C112, Vazyme, Nanjing, China). The recombinant vectors were, respectively, transformed into *Escherichia coli* strain BL21 (DE3). The His-HY5 and His-BRC1 recombinant proteins were separately expressed and purified using the Novagen pET Purification System (Madison, WI, USA) according to the manufacturer’s instructions. The probes were biotin end-labeled according to the instructions of the Biotin 3′ End DNA Labeling kit (Pierce, #89818, Thermo Fisher Scientific, Waltham) and annealed to a double-stranded probe DNA. EMSAs were performed according to the instructions of the Light Shift Chemiluminescent EMSA kit (Thermo Fisher Scientific, 20148). The DNA probes used in the EMSA are shown in *SI Appendix*, Table S5.

### Chromatin Immunoprecipitation (ChIP)-qPCR.

ChIP assay was carried out using the EpiQuik^TM^ Plant ChIP Kit (Epigentek, Farmingdale). Approximately 1.0 g of buds was harvested from *HY5*-3HA-OE and WT plants. Chromatin was immunoprecipitated with an anti-HA antibody (Abcam, ab18181); goat anti-mouse IgG antibody (Abcam, ab205719) was used as the negative control. ChIP-qPCR was performed with specific primers as shown in *SI Appendix*, Table S6.

### Dual-Luciferase Assay.

Dual-luciferase assays were performed as previously described (62, 63). The full-length coding region of *HY5* or *BRC1* was amplified and fused to the pGreen II 0029 62-SK(SK) vector, while the promoters of *BRC1, DET2, DWF, LOG4, CKX7, GA2ox4, and GA2ox5* were cloned into the pGreen II 0800-LUC (LUC) vector. The primers used are listed in *SI Appendix*, Table S7. *A. tumefaciens* strain GV3101 containing the SK and LUC constructs was resuspended in in-filtration buffer and adjusted to a concentration of OD_600 _= 0.7 to 0.8. Then, the mixture of the SK and LUC suspensions (10:1, v:v) was infiltrated into *Nicotiana benthamiana* leaves. The activities of firefly luciferase (LUC) and Renilla luciferase (REN) were assayed 3 d after infiltration by using a modulus luminometer (Promega). The LUC/REN value in the absence of HY5 or BRC1 protein was set as one, and the analyses were performed with six replicates. Measurements were carried out using a modulus luminometer (Promega).

### Yeast One-Hybrid Assay.

Y1H assay was performed using Matchmatch^TM^ Gold Yeast One-Hybrid System (Clontech) according to the manufacturer’s instructions. *LOG4, CKX7, GA2ox4,* and *GA2ox5* promoters were ligated into the pAbAi vector and the full-length coding region of *BRC1* was also amplified using specific primers (*SI Appendix*, Table S8) and fused to the pGADT7 vector. The linearized pAbAi constructs containing *LOG4, CKX7, GA2ox4, or GA2ox5* promoter were individually transformed into Y1HGold yeast strain. BRC1-AD, or an empty AD vector was transformed into the modified Y1HGold yeast strain. The transformed yeast cells were selected on SD/Leu- media supplemented with 100 ng mL^−1^ AbA.

## Statistical Analysis.

Statistical analyses were conducted with Statistics Package for Social Science (SPSS) statistical software (version. 19.0, SPSS, International Business Machines (IBM) Corporation, Armonk, NY, USA). The significance of treatment differences was analyzed using Student’s *t* test or Tukey’s test (*P* < 0.05), which is indicated in the figure legends.

## Supplementary Material

Appendix 01 (PDF)

Appendix 02 (PDF)

Dataset S01 (XLSX)

Dataset S02 (XLSX)

## Data Availability

All study data are included in the article and/or *SI Appendix*.
