# Supplementary material for: HY5 functions as a systemic signal by integrating BRC1-dependent hormone signaling in tomato bud outgrowth
Source: Proc Natl Acad Sci U S A. 2023 Apr 10;120(16):e2301879120. doi: 10.1073/pnas.2301879120 (PMC10120035; doi:10.1073/pnas.2301879120)
Supplement: Supplementary file 1 — Appendix 01 (PDF) [file pnas.2301879120.sapp1.pdf]

Supplementary figures

Article title: HY5 functions as a systemic signal by integrating BRC1-dependent hormone signaling in tomato bud outgrowth

The following Supporting tables is available for this article:

Figure S1 Bud outgrowth of wild type (WT) plants in response to changes in red to far red (R/FR) ratio.

Figure S2 Bud outgrowth of WT, *dwf* and *bzr1* plants in response to changes in red to far red (R/FR) ratio.

Figure S3 Photoreceptors participate in the regulation of bud outgrowth in tomato.

Figure S4 Bud outgrowth of WT*, HY5*-RNAi, *hy5* mutant and *HY5*-OE plants.

Figure S5 Suppression of *HY5* transcript compromised *CRY1a* overexpression-induced bud outgrowth in tomato.

Figure S6 *BRANCHED1* (*BRC1*) acts downstream of HY5 to regulate bud outgrowth in tomato.

FigureS7 Schematic illustration of the isolation of *brc1* mutants and bud outgrowth of WT, *brc1 #5, brc1 #9* plants.

Figure S8 Bud outgrowth in *hy5* and *brc1* mutants as influenced by light quality.

Figure S9 Schematic representation of the G-, C- , Z- and A-BOX motifs in the *DET2*, *DWF* and *BRC1* promoters and the vector construction principle used in the dual-luciferase assays.

Figure S10 Cytokinin (CK) and gibberellin (GA) pathways are involved in the BRC1 inhibition of branching in tomato.

Figure S11 BRC1-inhibited bud outgrowth is not dependent on the biosynthesis of abscisic acid (ABA) and indole-3-acetic acid (IAA) in tomato.

Figure S12 Schematic representation of the motifs in the *LOG4*, *CKX7*, *GA2ox4*, *GA2ox5* promoters and the vector construction principle used in the dual-luciferase assays.

Figure S13 The transgenic tomato of pBRC1-*LOG4* or pBRC1-*CKX7* showed elevated accumulation of LOG4 or CKX7 protein in the lateral buds.

Figure S14 *LOG4*, *CKX7*, *GA2ox4*, and *GA2ox5* act downstream of BRC1 in the regulation of bud outgrowth in tomato.

Figure S15 The diurnal changes in the *HY5* and *BZR1* transcript in the lateral buds of WT plants.

Figure S16 The sucrose content in lateral buds and the role of sucrose in bud outgrowth.

Figure S17 The mobile HY5 protein is a systemic signal to regulate shoot branching in tomato.

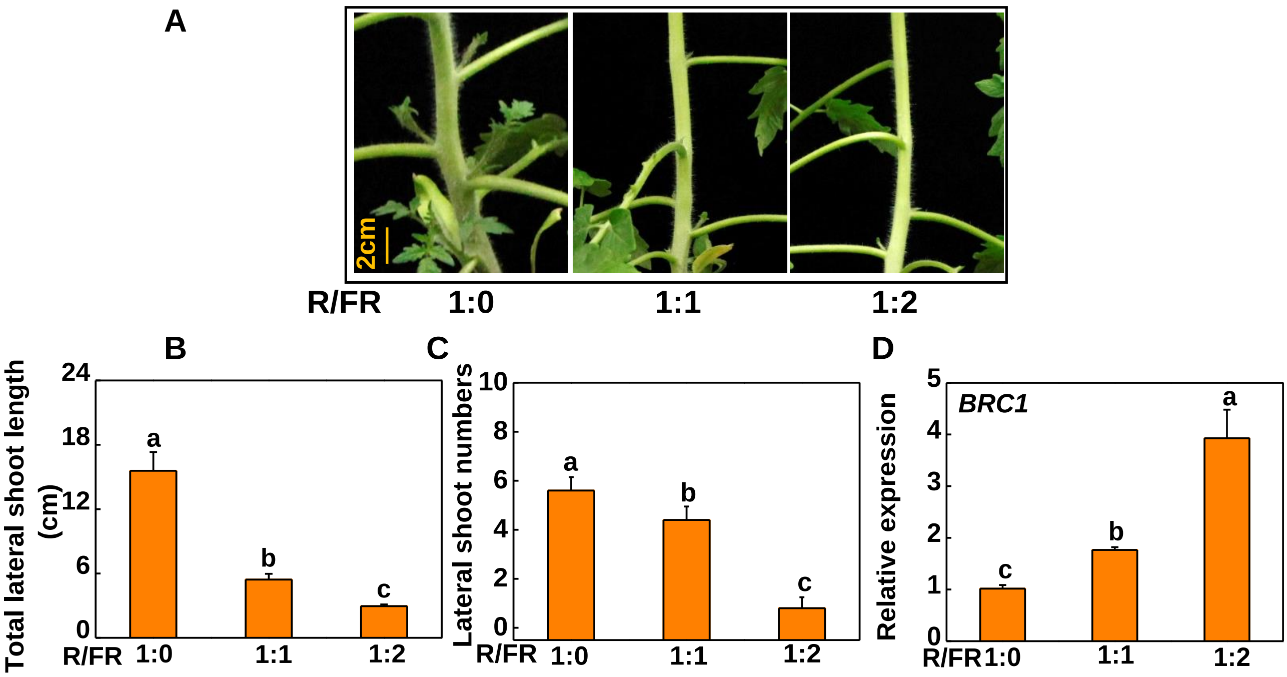

Fig. S1. Bud outgrowth of wild type (WT) plants in response to changes in red to far red (R/FR) ratio. (A) Bud outgrowth phenotypes. (B) The total lateral shoot length at 7d after the first application of R/FR treatment. (C) The lateral shoot numbers per plant. (D) Transcript of *BRC1* in the lateral bud from the 4th stem node at 6 h after the R/FR treatment. For the qRT-PCR analysis, *ACTIN2* and *UBI3* were used as reference genes, and the gene expression at R/FR=1:0 was defined as 1. Data are presented as the means of replicates ± standard deviation (SD). n=12-15 in (B, C) and n=3-4 in (D), respectively. Different letters indicate significant differences (P<0.05) according to Tukey's test.

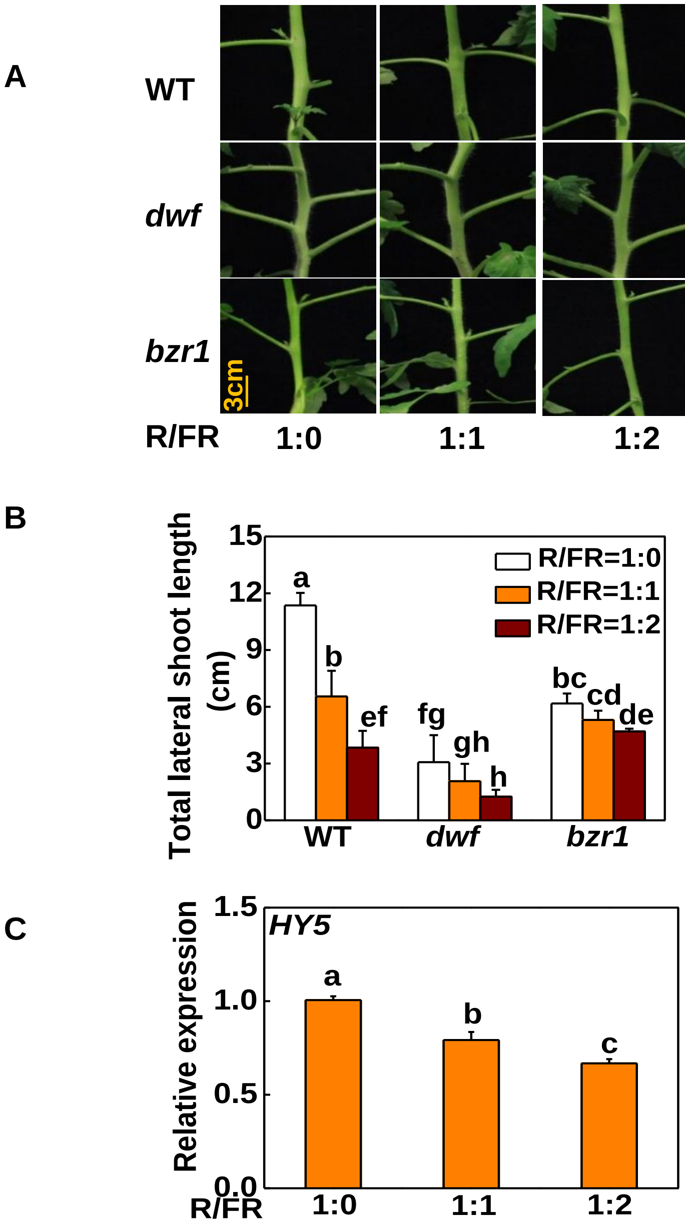

Fig. S2. Bud outgrowth of WT, *dwf* and *bzr1* plants in response to changes in red to far red (R/FR) ratio. (A) Bud outgrowth phenotypes at 7d after the first application of R/FR treatment. (B) The total lateral shoot length of WT plant, *dwf* and *bzr1* mutants plants at 7d after the first application of R/FR treatment. (C) Transcript of *HY5* in the lateral buds from the 4th stem node of WT plants in response to changes in red to far red (R/FR) ratio. *ACTIN2* and *UBI3* were used as reference genes, and the gene expression under R/FR=1:0 was defined as 1. Samples were taken at 6 h after the light quality treatment. Data are presented as the means of replicates ± standard deviation (SD). n=12-15 in (B) and n=3-4 in (C), respectively. Different letters indicate significant differences (P<0.05) according to Tukey's test.

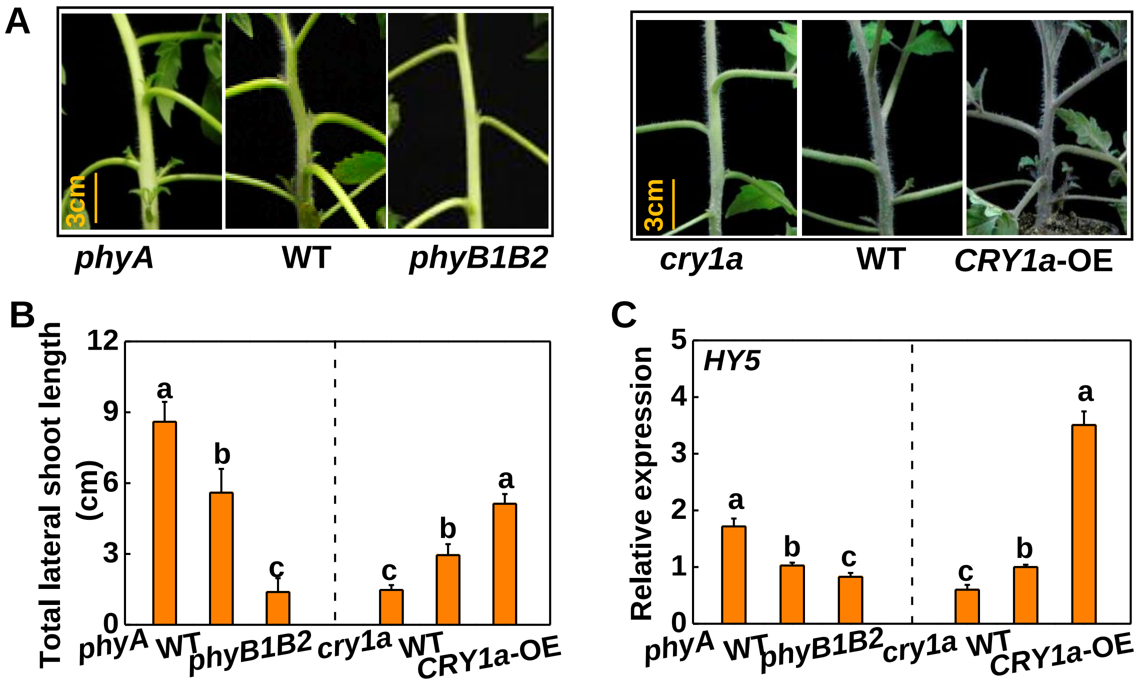

Fig. S3. Photoreceptors participate in the regulation of bud outgrowth in tomato. (A) Bud outgrowth phenotypes of *phyA*, WT, *phyB1B2* plants and *cry1a*, WT and *CRY1a*-OE plants under WL. (B) The total lateral shoot length of *phyA*, WT, *phyB1B2* plants and *cry1a*, WT and *CRY1a*-OE plants under WL. (C)Transcript of *HY5* in the lateral buds from the 4th stem node of *phyA*, WT, *phyB1B2* plants and *cry1a*, WT and *CRY1a*-OE plants under WL. *ACTIN2* and *UBI3* were used as reference genes, and the gene expression in WT was defined as 1. All plants at 8-leaf stage were grown under WL. Samples were taken at 12 AM. Data are presented as the means of replicates ± standard deviation (SD). n=12-15 in (B) and n=3-4 in (C), respectively. Different letters indicate significant differences (P<0.05) according to Tukey's test.

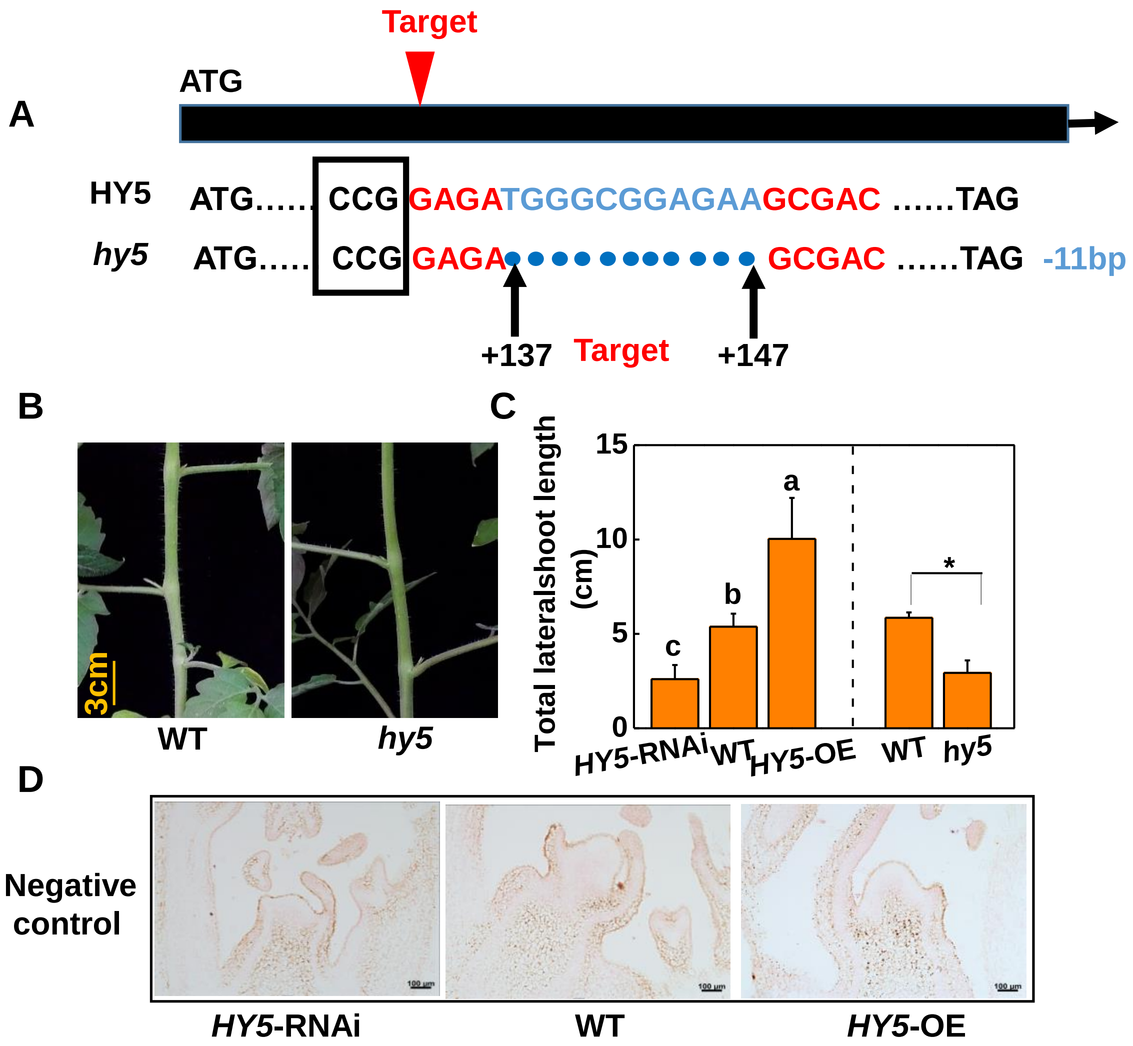

Fig. S4. Bud outgrowth of WT, *HY5*-RNAi, *hy5* mutant and *HY5*-OE plants. (A) Schematic illustration of the sgRNA target site (red arrows) in wild type (WT) and *hy5* from CRISPR/Cas9 T2 mutant lines. Sequence analysis indicated that *hy5* mutant had an 11 bases deletion at 137-147 position in the *HY5* open reading frame (ORF), which leads to frame shift and the generation of a premature stop codon, TAG. Red font presents sgRNA target sequence, and black box presents protospacer-adjacent motif (PAM) sequences. (B) Bud outgrowth phenotypes of WT, *hy5* plants under WL. (C) The total lateral shoot length. All plants at 8-leaf stage growth under WL were used for experiment. Data are presented as the means of replicates ± standard deviation (SD). n=12-15 in (C). Different letters or * indicate significant differences (P<0.05) according to Tukey's test or Student's t test (P < 0.05), respectively. (D) In *situ* hybridization of negative control in axillary buds. Three independent biological samples were performed, with similar results.

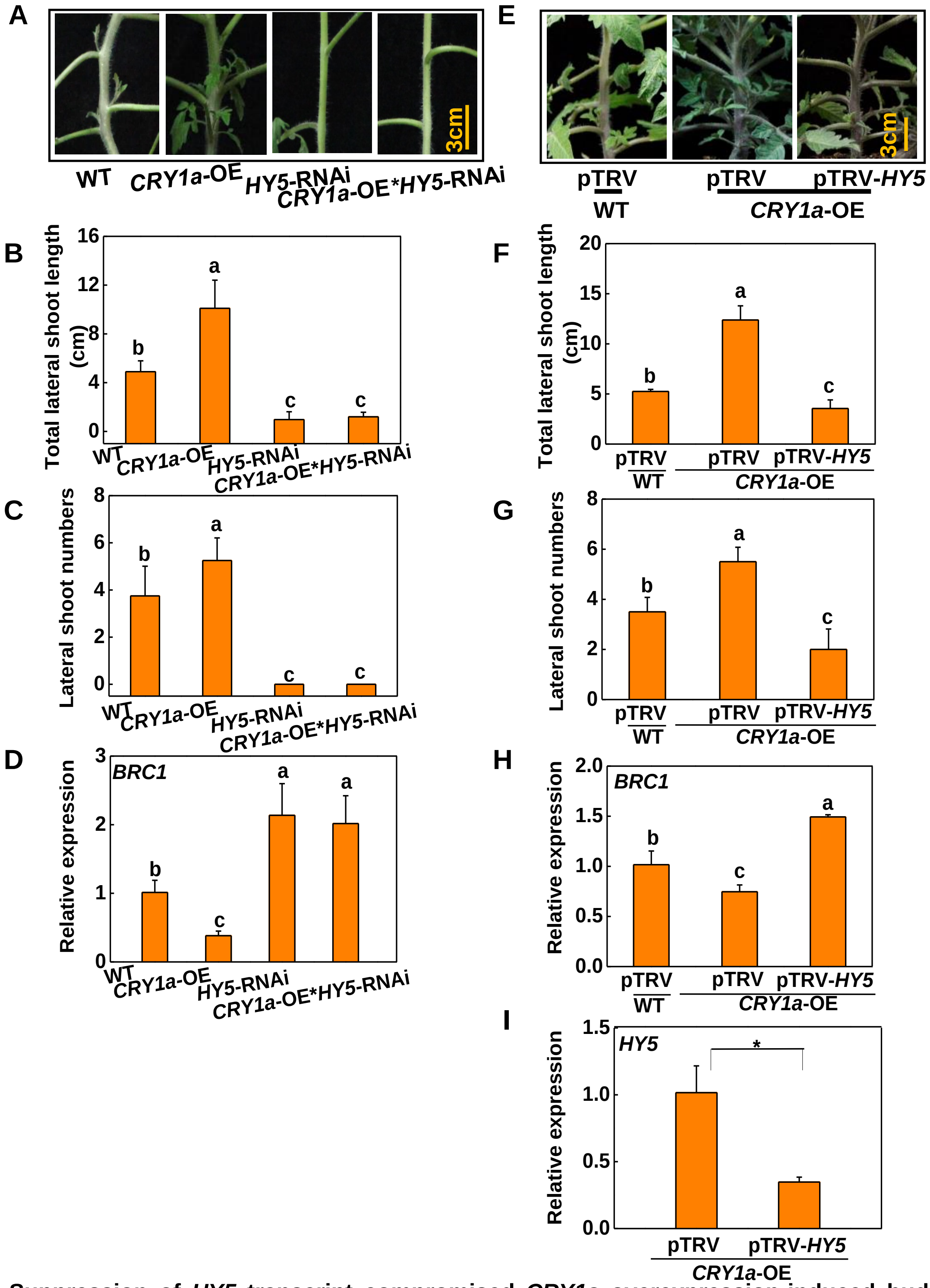

Fig. S5. Suppression of *HY5* transcript compromised *CRY1a* overexpression-induced bud outgrowth in tomato. (A, E) Bud outgrowth phenotypes. (B,F) The total lateral shoot length. (C,G) The lateral shoot numbers per plant. (D,H) Relative transcript of *BRANCHED1* (*BRC1*) in the lateral buds from the 4th stem node. (I) VIGS efficiency analysis. For the qRT-PCR analysis, *ACTIN2* and *UBI3* were used as reference genes, and the gene expression in the WT-pTRV (F-H) or *CRY1a-OE*-pTRV (I) was defined as 1. All plants at 8-leaf stage growth under WL were used for experiment. Samples were taken at 12 AM. Data are presented as the means of replicates ± standard deviation (SD). n=12-15 in (B,F,C,G) and n=3-4 in (D,H,I), respectively. Different letters or * indicate significant differences (P<0.05) according to Tukey's test or Student's t test (P < 0.05), respectively.

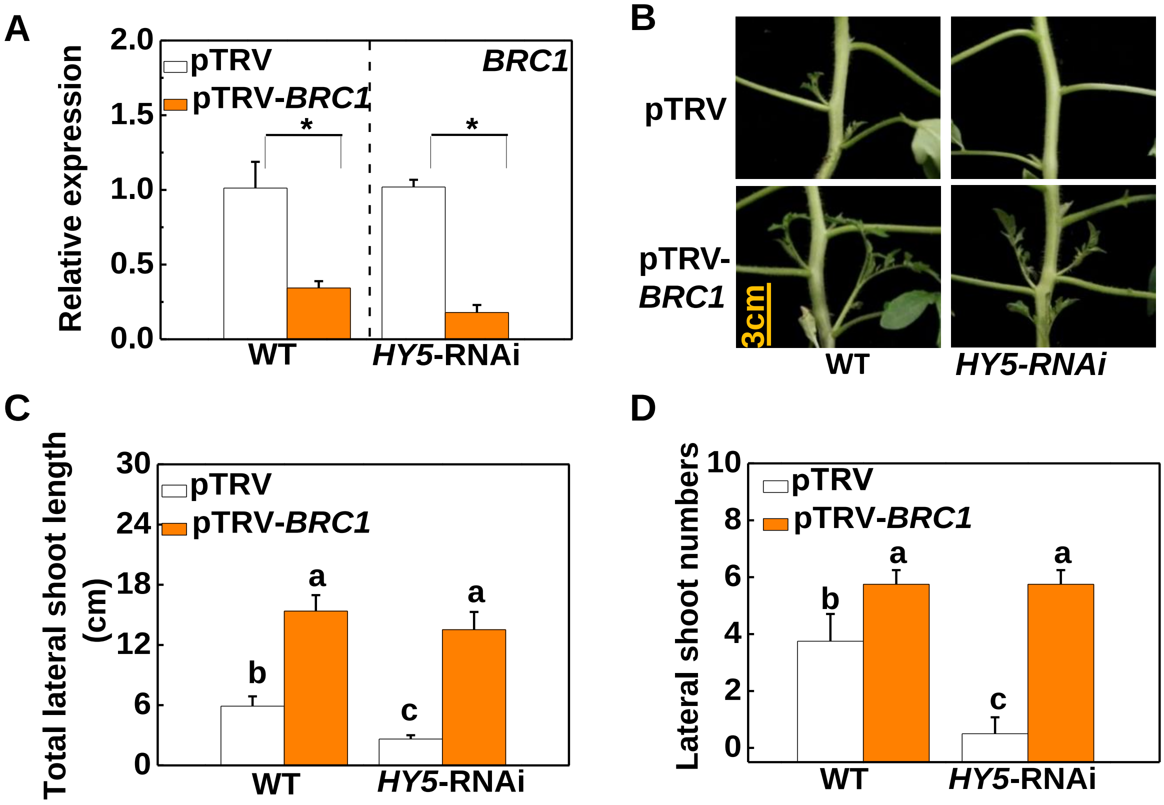

Fig. S6. *BRANCHED1* (*BRC1*) acts downstream of HY5 to regulate bud outgrowth in tomato. (A) VIGS efficiency analysis. *ACTIN2* and *UBI3* were used as reference genes, and the gene expression in the pTRV was defined as 1. (B) Phenotype. (C) The total lateral shoot length.(D) The lateral shoot numbers per plant. For the VIGS experiments, the infiltrated plants were grown in a growth room under WL (12 h photoperiod) at 23/20 ° C . Plants at 8-leaf stage were used for experiment. Data are presented as the means of replicates ± standard deviation (SD). n=3-4 in (A) and n=12-15 in (C,D), respectively. Different letters or * indicate significant differences (P<0.05) according to Tukey's test or Student's t test (P < 0.05), respectively.

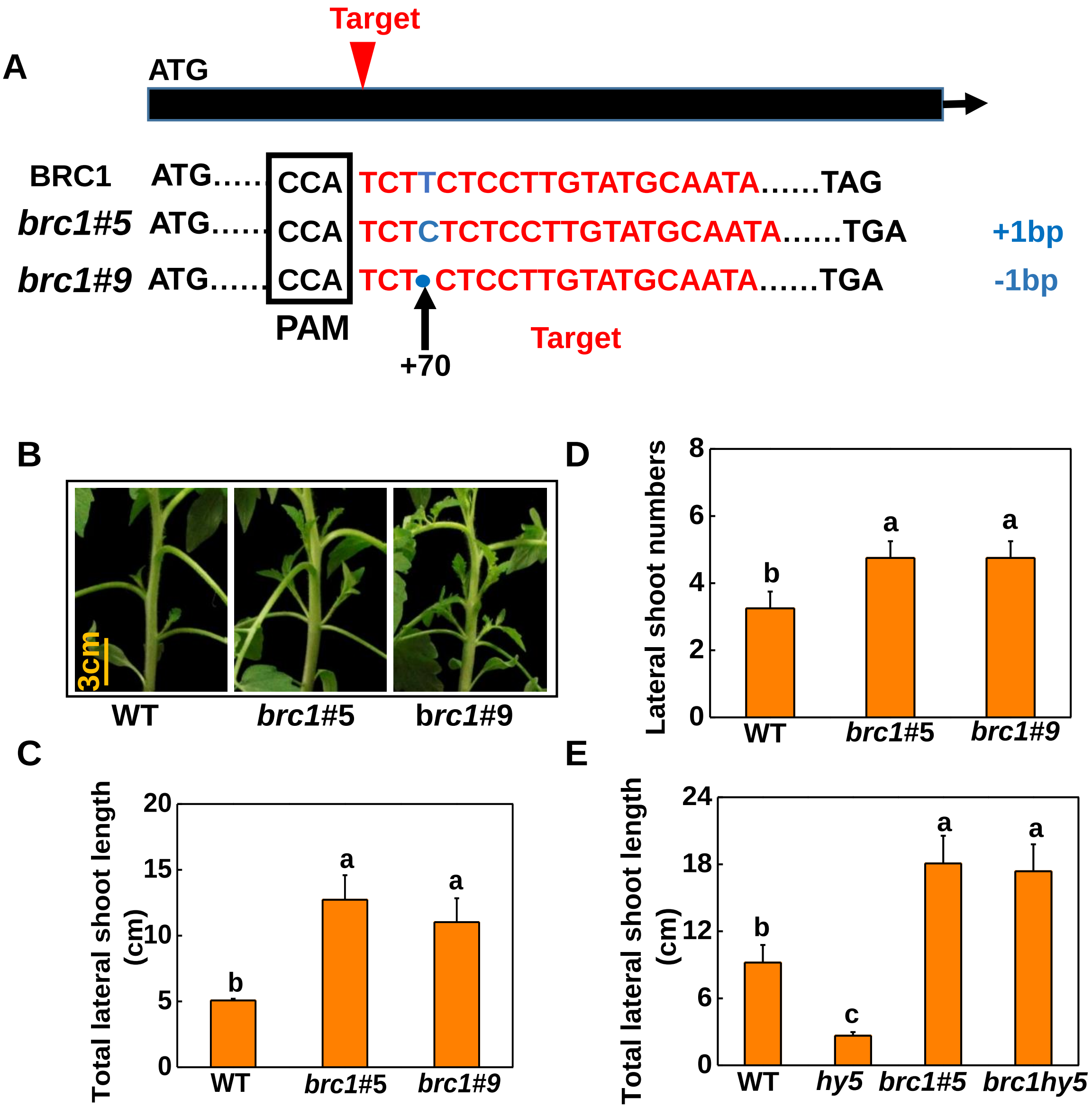

Fig. S7. Schematic illustration of the isolation of *brc1* mutants and bud outgrowth of WT, *brc1 #5, brc1 #9* plants. (A) Schematic illustration of the sgRNA target site (red arrows) in wild type (WT) and two alleles of *brc1 #5* and *brc1 #9* from CRISPR/Cas9 T2 mutant lines. Sequence analysis indicated that *brc1* #5 and *brc1* #9 mutants contain a C insertion or T deletion at 70 position in the *BRC1* open reading frame (ORF), which leads to frame shift and the generation of a premature stop codon, TGA. Red font and black box indicate sgRNA target sequence and the protospacer-adjacent motif (PAM) sequences, respectively. (B) Bud outgrowth phenotypes of WT, *brc1 # 5* and *brc1 # 9* plants. (C, D) The total lateral shoot length and the lateral shoot numbers in WT plant and *brc1* mutants. (E) The total lateral shoot length of WT,*hy5, brc1# 5* and *brc1hy5* plants. All these plants were grown under WL. Data are presented as the means of replicates ± standard deviation (SD). n=12-15. Different letters indicate significant differences (P<0.05) according to Tukey's test.

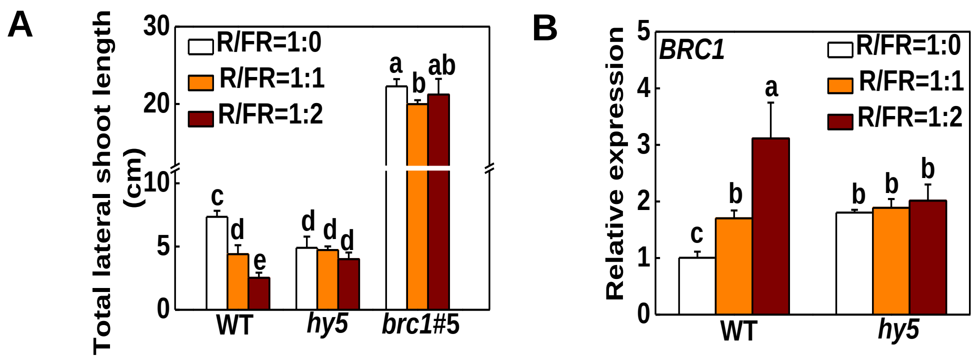

Fig. S8. Bud outgrowth in *hy5* and *brc1* mutants as influenced by light quality. (A) The total lateral bud length of WT, *hy5* and *brc1#5* plants in response to the changes in red to far red (R/FR) ratio. (B) Transcript of *BRC1* in the lateral buds from the 4th stem node of WT, and *hy5* mutant at 6 h after the R/FR treatment. *ACTIN2* and *UBI3* were used as reference genes, and the gene expression in WT plant under the R/FR at 1:0 was defined as 1. Data are presented as the means of three replicates ± standard deviation (SD). n=12-15 in (A) and n=3-4 in (B), respectively. Different letters indicate significant differences (*P*<0.05) according to Tukey's test.

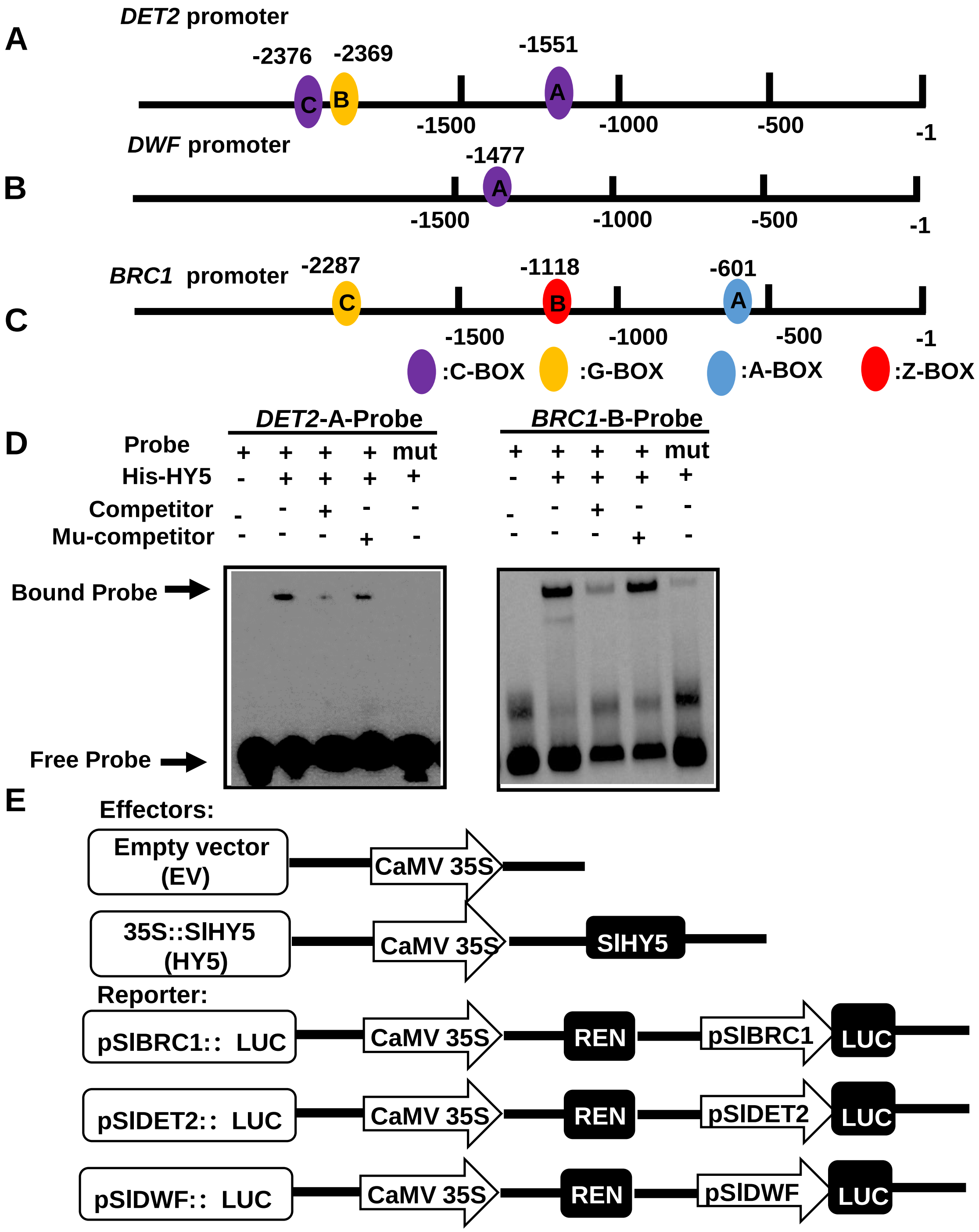

Fig. S9. Schematic representation of the G-, C- , Z- and A-BOX motifs in the *DET2*, *DWF* and *BRC1* promoters and the vector construction principle used in the dual-luciferase assays. (A,B,C) Schematic representation of the A-BOX (TACGTA), G-BOX (CACGTG), Z-BOX （TACGTG）and C-BOX (GTCANN) in the promoters of *DET2, DWF* and *BRC1*. Upstream sequences (within 3 kb) of the indicated genes are shown. The translational start site (ATG) is shown as position +1. The G-, C- , Z- and A-BOX in the promoters are presented as yellow, purple, red and blue respectively. (D) EMSA assay to test the binding ability of HY5 to the *DET2* and *BRC1* promoters. (E) Vector construction principle of dual-luciferase reporter gene.

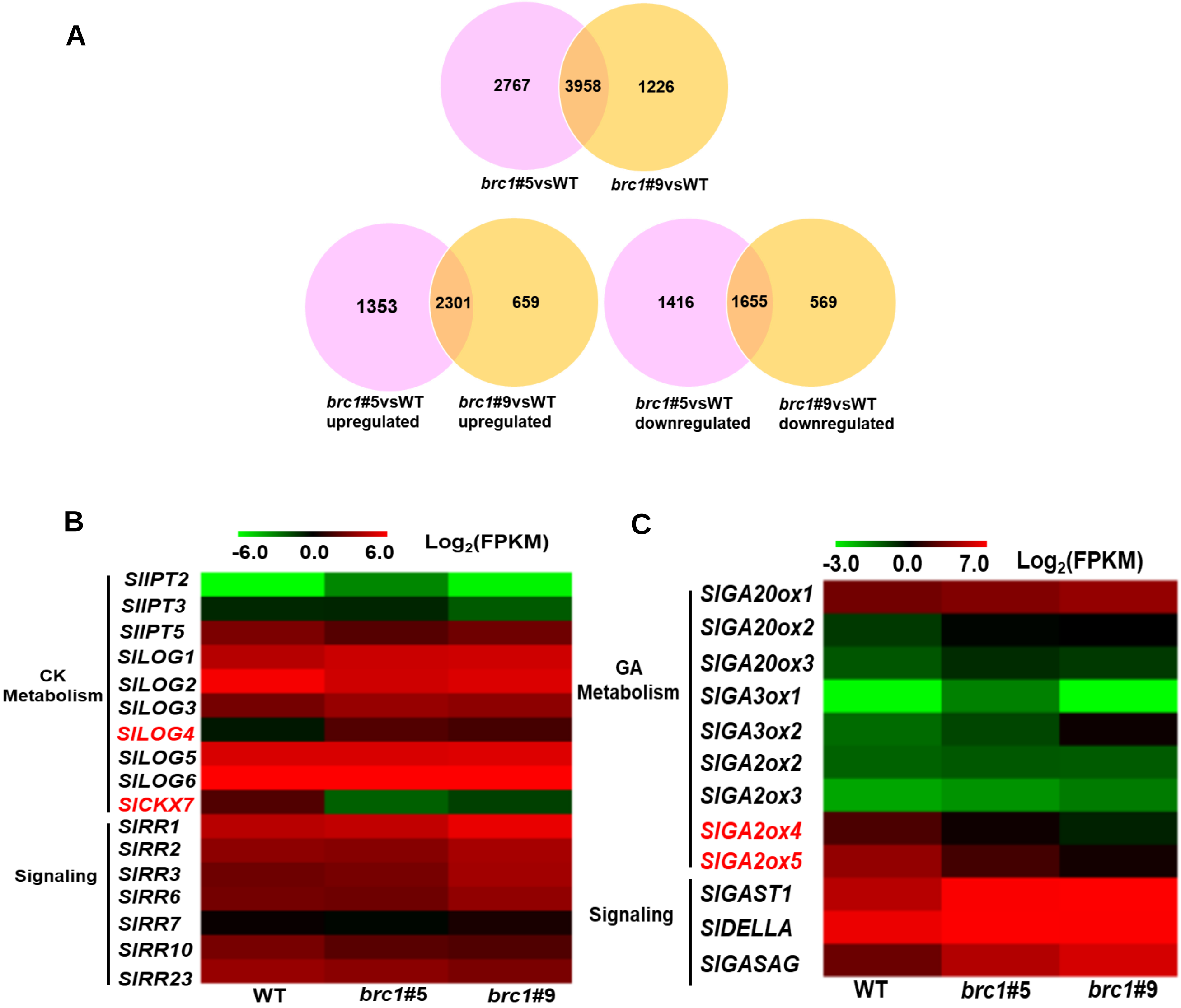

Fig. S10. Cytokinin (CK) and gibberellin (GA) pathways are involved in the BRC1 inhibition of branching in tomato. (A) Differentially expressed genes in the lateral buds of *brc1* mutants and WT plant. (B) Expression of representative CK-responsive genes in the RNA-seq experiments. (C) Expression of representative GA-responsive genes in the RNA-seq experiments. Data are presented as the means of three replicates.

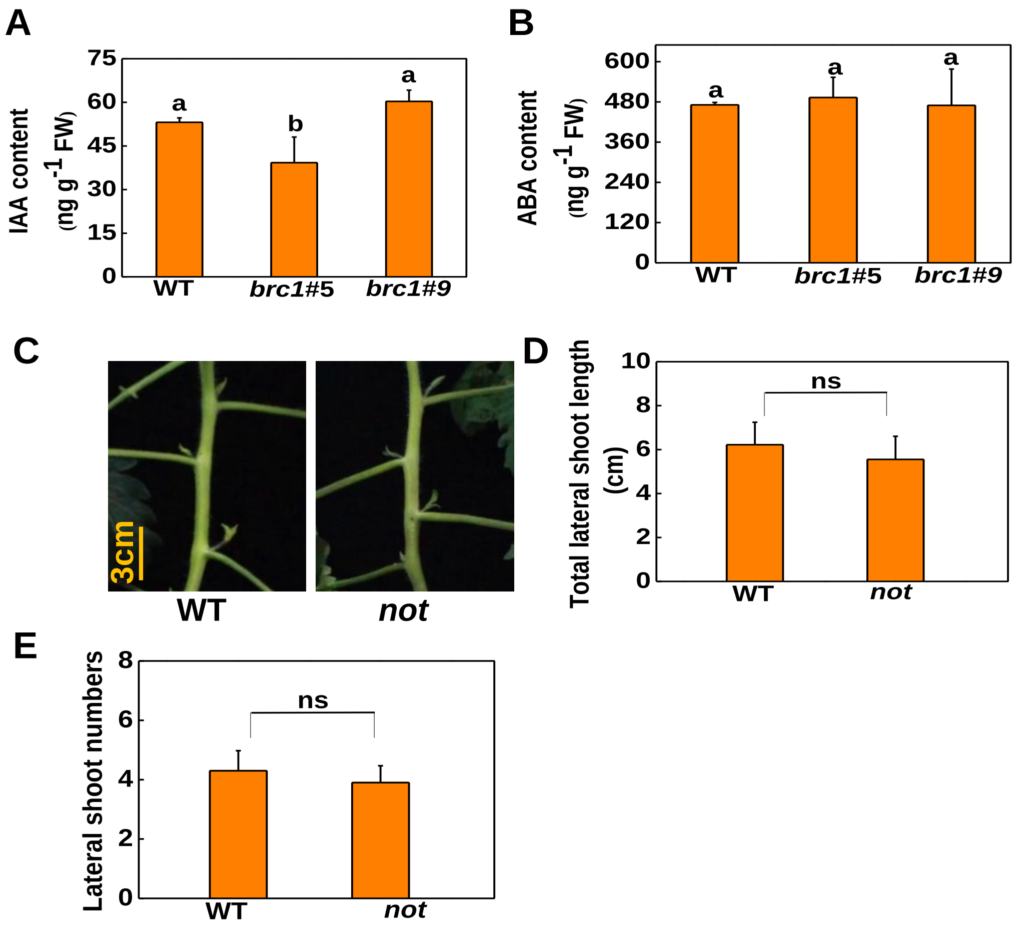

Fig. S11. BRC1-inhibited bud outgrowth is not dependent on the biosynthesis of abscisic acid (ABA) and indole-3-acetic acid (IAA) in tomato.(A, B) IAA and ABA content in the lateral buds. (C, D) Bud outgrowth phenotypes and the total lateral shoot length of WT and *not* plants*.* (E) Lateral shoot numbers per plant of WT and *not* plants. All plants were grown under WL. Samples were taken at 12 AM. Data are presented as the means of replicates ± standard deviation (SD). n=3-4 in (A,B) and n=12-15 in (D,E), respectively. Different letters or * indicate significant differences (P<0.05) according to Tukey's test or Student's t test (P < 0.05), respectively.

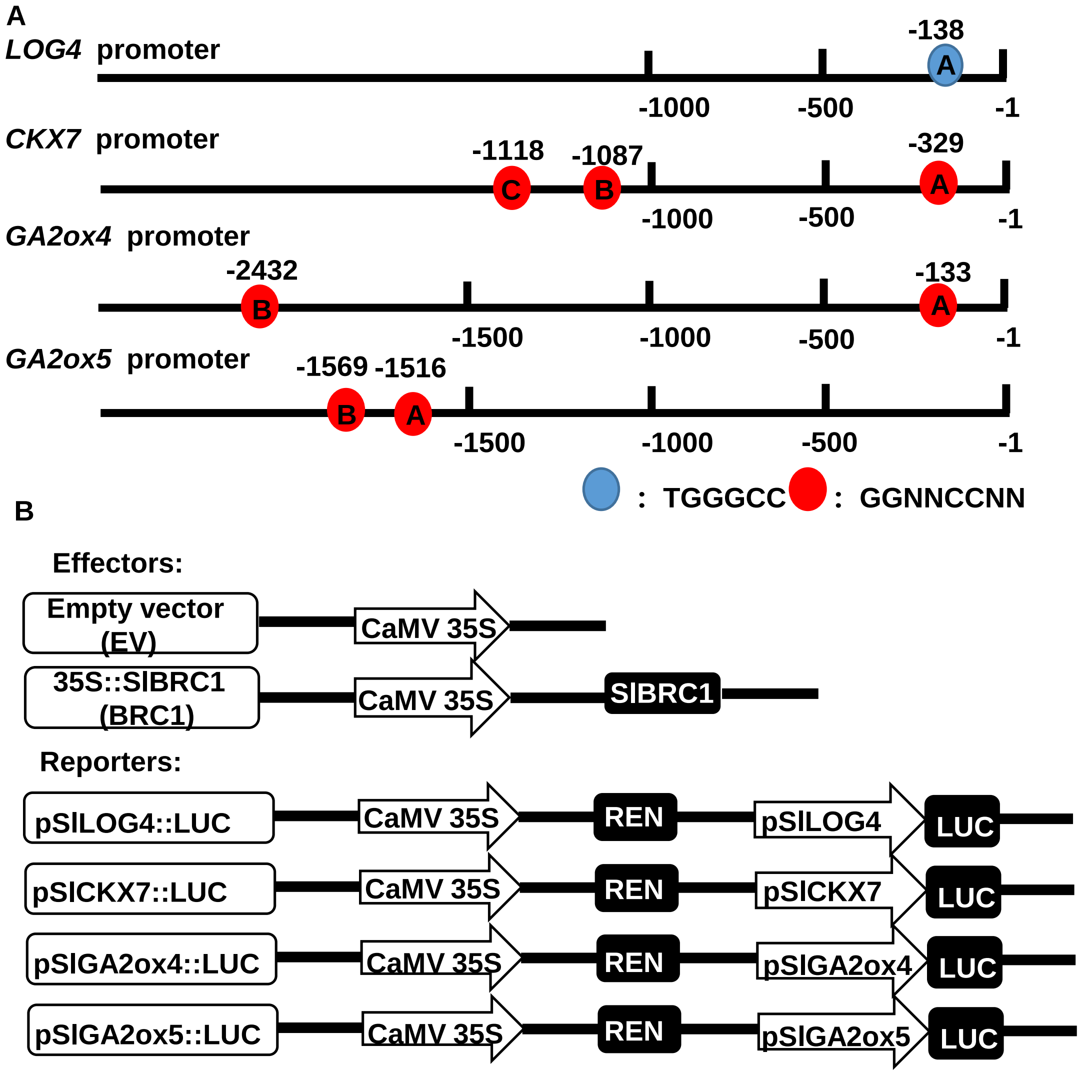

Fig. S12. Schematic representation of the motifs in the *LOG4*, *CKX7*, *GA2ox4*, *GA2ox5* promoters and the vector construction principle used in the dual-luciferase assays. (A) Schematic representation of the putative elements (GGNNCCNN, TGGGCC) in the promoters of *LOG4, GA2ox4, GA2ox5* and *CKX7*. The translational start site (ATG) is shown as position +1. The putative elements GGNNCCNN, TGGGCC in the promoters are presented as red and blue respectively. (B) Vector construction principle of dual-luciferase reporter gene.

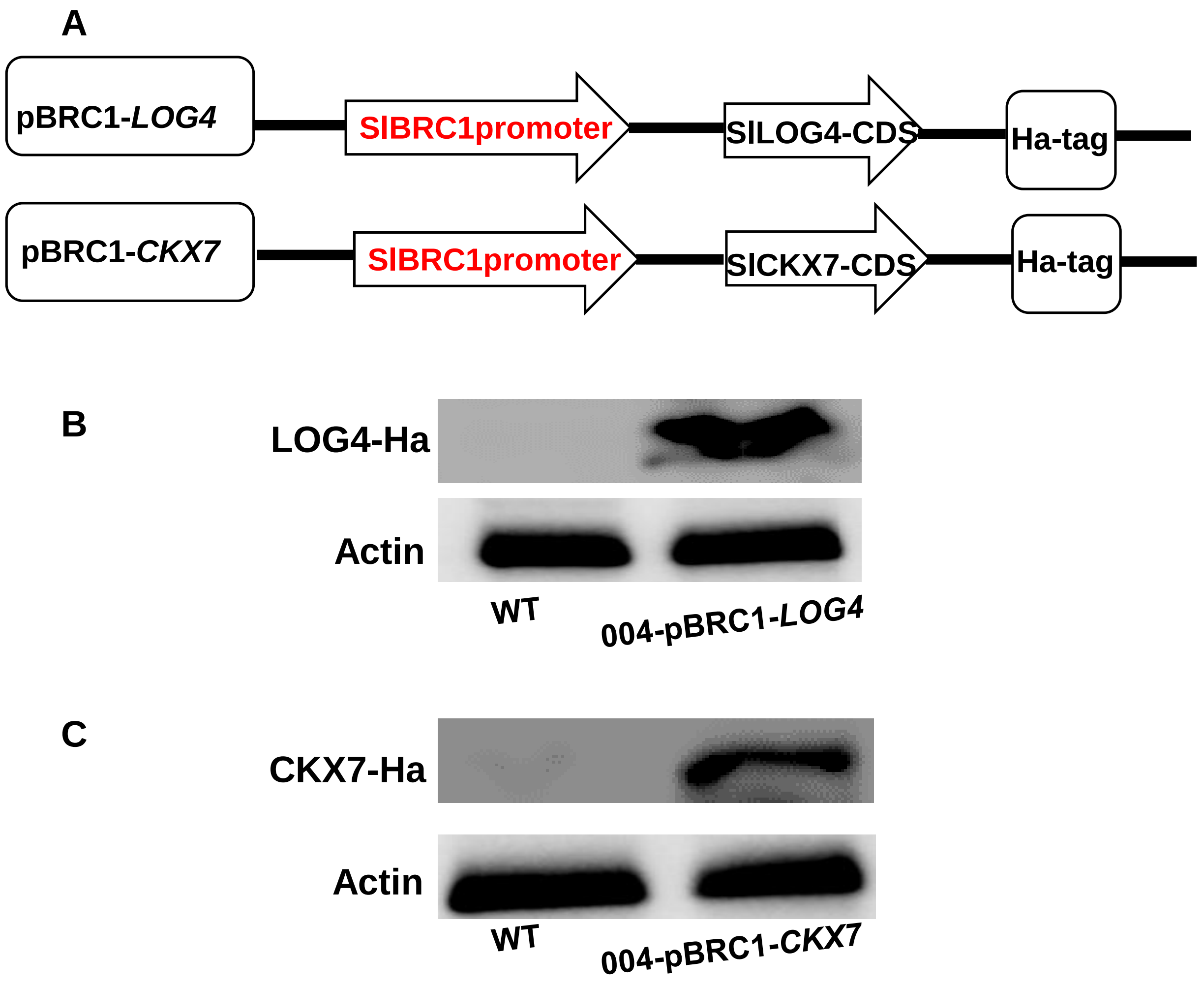

Fig. S13. The transgenic tomato of pBRC1-*LOG4* or pBRC1-*CKX7* showed elevated accumulation of LOG4 or CKX7 protein in the lateral buds. (A) Schematic illustration of the isolation of pBRC1-*LOG4* and pBRC1-*CKX7* plants. (B,C) The protein of LOG4 and CKX7 in the lateral buds of pBRC1-*LOG4* and pBRC1-*CKX7* plants. Actin was used as a loading control for the western blot analysis.

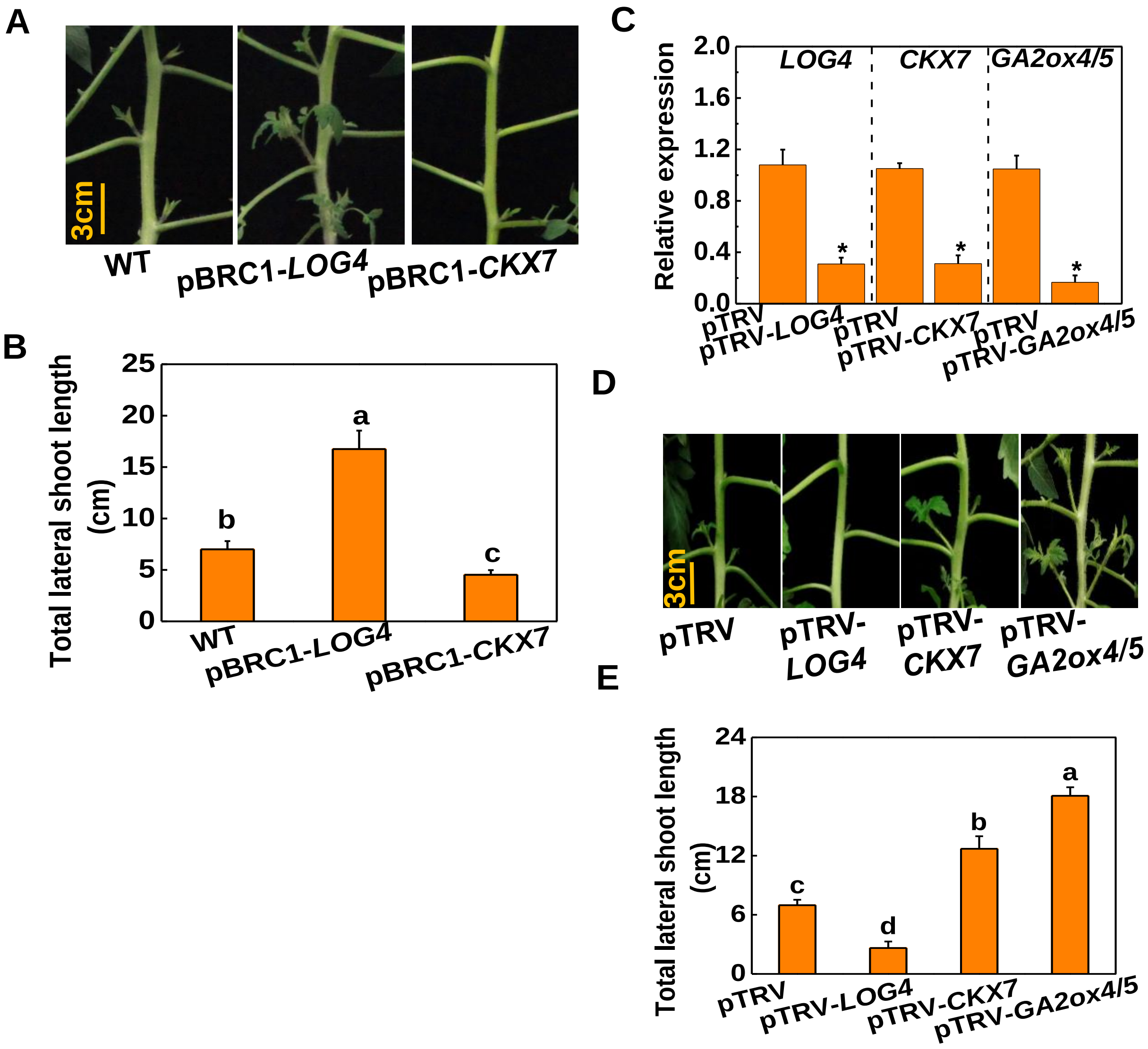

Fig. S14. *LOG4*, *CKX7*, *GA2ox4*, and *GA2ox5* act downstream of BRC1 in the regulation of bud outgrowth in tomato. (A,B) Elevated expression of *SlLOG4* or *SlCKX7* driven by the *SlBRC1* promoter (pBRC1-*LOG4* or pBRC1-*CKX7*), resulted in the changes of bud outgrowth phenotypes and the total lateral shoot length. (C) Efficiency of silencing *LOG4, CKX7*, and co-silencing *GA2ox4* and *GA2ox5. ACTIN2* and *UBI3* were used as reference genes, and the gene expression in the pTRV was defined as 1 (D, E) Bud outgrowth phenotypes and the total lateral shoot length of plants after silencing of *LOG4, CKX7*, and co-silencing *GA2ox4* and *GA2ox5*, respectively. All plants were grown under WL. Data are presented as the means of replicates ± standard deviation (SD). n=4 in (C) and n=12-15 in (B,E), respectively. Different letters or * indicate significant differences (P<0.05) according to Tukey's test or Student's *t* test (P < 0.05), respectively.

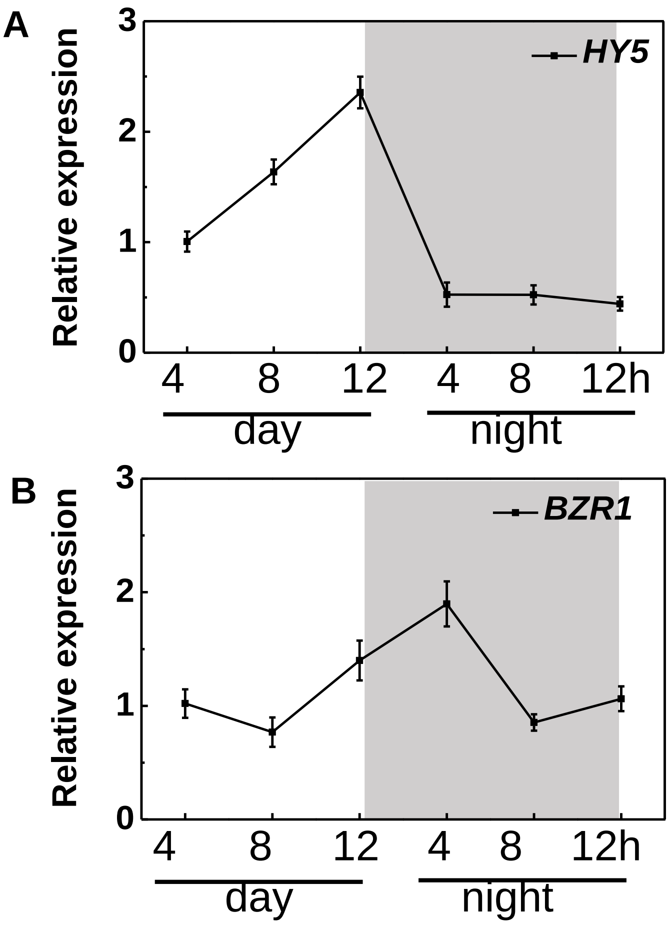

Fig. S15. The diurnal changes in the *HY5* and *BZR1* transcript in the lateral buds of WT plants. WT plants at 8-leaf stage were grown under WL. The lateral buds from the 4th stem node were collected at 4 h interval. Black box indicates dark time. *ACTIN2* and *UBI3* were used as reference genes, and the gene expression in WT plant at 4h in the day (12 AM) was defined as 1. Data are presented as the means of four replicates ± standard deviation (SD).

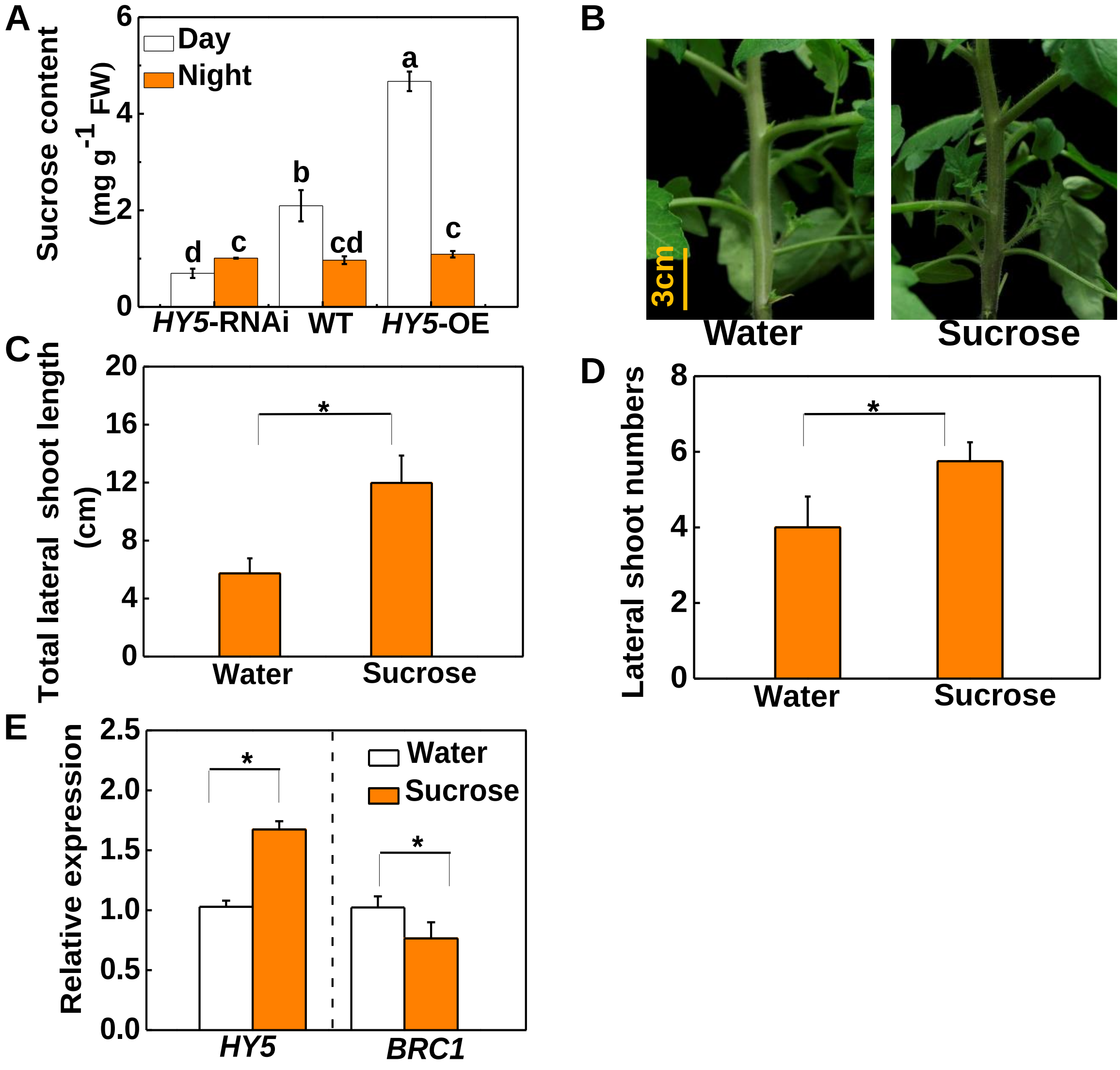

Fig. S16. The sucrose content in lateral buds and the role of sucrose in bud outgrowth. (A) The sucrose content in the lateral buds as influenced by HY5 and light. (B,C,D) Effects of foliar application of sucrose on bud outgrowth phenotypes. (E) Effects of foliar application of sucrose on the transcript of *HY5* and *BRC1* in the lateral buds. Plants at 8-leaf stage grown under WL were sprayed with 15 mL of sucrose solution at 20 mM. Samples were taken at 8 AM and 8 PM in (A) and 12AM in (E). Data are presented as the means of replicates ± standard deviation (SD). n=3-4 in (A,E) and n=12-15 in (C, D), respectively. Different letters or * indicate significant differences (P<0.05) according to Tukey's test or Student's t test (P < 0.05), respectively.

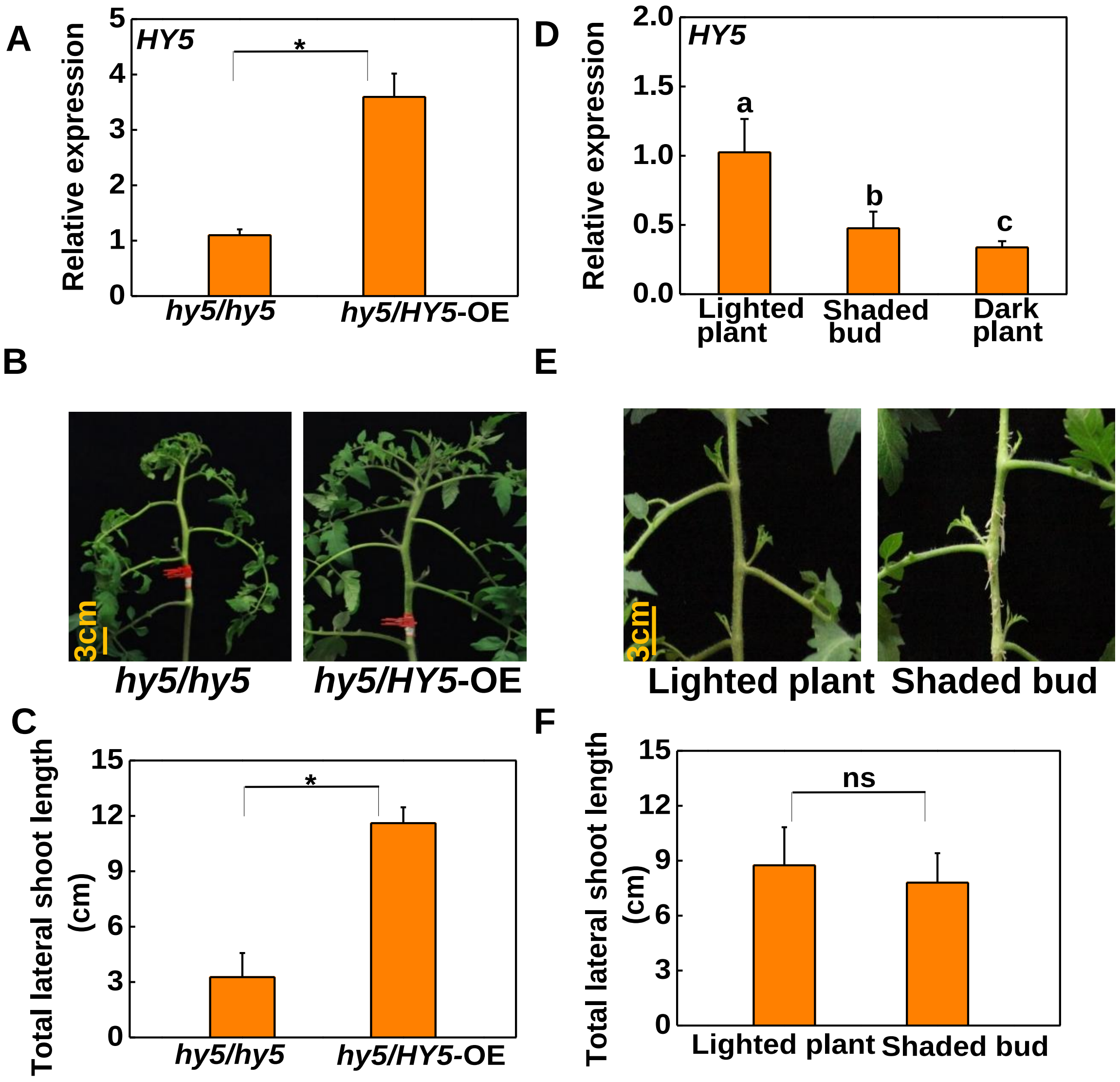

Fig. S17. The mobile HY5 protein is a systemic signal to regulate shoot branching in tomato. (A) Expression of *HY5* in the scion lateral buds on hy5 scion in the grafts. *ACTIN2* and *UBI3 were used as* the reference genes, and the gene expression in *hy5/hy5* was defined as 1. (B,C) Bud outgrowth phenotypes and the total lateral shoot length of the grafts. *HY5*-RNAi plants with 4 leaves were self-grafted (*hy5*/*hy5*) or grafted onto *HY5*-OE plants (*hy5*/*HY5*-OE) with 4 leaves. (D) Expression of *HY5* in the buds from the lighted plants, shaded bud and from the dark plant. (E,F) Bud outgrowth phenotypes and the total lateral shoot length of WT plants with the axillary buds shaded or not shaded with tin foil. Plants were grown under 12 h light cycle under WL. Samples were taken at 12 AM in (A,D).Data are presented as the means of replicates ± standard deviation (SD). n=4 in (A,D) and n=12-15 in (C, F), respectively. Different letters or * indicate significant differences (P<0.05) according to Tukey's test or Student's t test (P < 0.05), respectively.
