# Supplementary material for: HY5 functions as a systemic signal by integrating BRC1-dependent hormone signaling in tomato bud outgrowth
Source: Proc Natl Acad Sci U S A. 2023 Apr 10;120(16):e2301879120. doi: 10.1073/pnas.2301879120 (PMC10120035; doi:10.1073/pnas.2301879120)
Supplement: Supplementary file 2 — Appendix 02 (PDF) [file pnas.2301879120.sapp2.pdf]

Supplementary tables

Article title: HY5 functions as a systemic signal by integrating BRC1-dependent hormone signaling in tomato bud outgrowth

The following Supporting tables is available for this article:

Table S1 PCR primer sequences used for vector construction.

Table S2 PCR primer sequences used for vector construction in VIGS.

Table S3 List of primer sequences used for the qRT-PCR analysis.

Table S4 PCR primer sequences used for vector construction of pET-32a-His-BRC1.

Table S5 Probes used in the electrophoretic mobility shift assays (EMSA).

Table S6 Primers used for the ChIP-qPCR assays.

Table S7 PCR primer sequences used for vector construction in the dual-luciferase assays.

Table S8 Primers used for pAbAi-baits and AD-prey constructs.

Table S1 PCR primer sequences used for vector construction.

ID	Primer (5'-3')
pBRC1-LOG4-F	acagctactccacaaggcgcgccATGGGTAATTTCAAGAAAATTTGTGT
pBRC1-LOG4-R	aacatcgtatgggtaggtaccACTTGTTGTCACAGATTCATCAGTGTT
pBRC1-CKX7-F	acagctactccacaaggcgcgccATGAAGTCACCAACCCAATTTTTC
pBRC1-CKX7-R	aacatcgtatgggtaggtaccTTCATGTTCACAGTCTGCCGTC

Table S2 PCR primer sequences used for vector construction in VIGS.

ID	Primer (5'-3')
VIGS-HY5-EcoRI-F	gtgagtaaggttaccgaattcATGCAAGAGCAAGCGACGA
VIGS-HY5-BamHI-R	cgtgagctcggtaccggatccTGCTGATACTCTATTTCTCAACAATCTT
VIGS-BRC1-XbaI-F	aaggttaccgaattctctagaATGTATCCTCCAAGCAACAATAACTG
VIGS-BRC1-BamHI-R	cgtgagctcggtaccggatccGCTACTCATAACATCATCATCTCCTTT
VIGS-LOG4-EcoRI-F	gtgagtaaggttaccgaattcACTAATGGGATTAGTTTCTCAAGCTG
VIGS-LOG4-BamHI-R	cgtgagctcggtaccggatccCCAAGCAAGCAATCATAATATCCA
VIGS-CKX7-EcoRI-F	gtgagtaaggttaccgaattcATGAAGTCACCAACCCAATTTTTC
VIGS-CKX7-BamHI-R	cgtgagctcggtaccggatccAGTACGGATTTCGAAGACATGTTTT
VIGS-GA2ox4- EcoRI-F	gtgagtaaggttaccgaattcAAATGGCGATATCGGTTGGG
VIGS-GA2ox4- BamHI-R	cgtgagctcggtaccggatccAAATTTTTGGCATTGAATTCTTGA
VIGS-GA2ox5-EcoRI-F	gtgagtaaggttaccgaattcGACAAAGTGGCGATATCGGTTG
VIGS-GA2ox5-BamHI-R	cgtgagctcggtaccggatccTTTTGGCATTGAATTCTTGAATATC

Table S3 List of primer sequences used for the qRT-PCR analysis.

Accession number	Gene	Primer (5'-3')
Solyc11g005330	RT-ACTIN2-F	TGTCCCTATTTACGAGGGTTATGC
	RT-ACTIN2-R	CAGTTAAATCACGACCAGCAAGAT
Solyc01g056940	RT-UBI3-F	GCCGACTACAACATCCAGAAGG
	RT-UBI3-R	TGCAACACAGCGAGCTTAACC
Solyc06g069240	RT-BRC1-F	ACACAGCAAGATCAACACCG
	RT-BRC1-R	GCTGGCCTTATCGAATCCCA
Solyc01g005680	RT-LOG4-F	CAGCTACATCACCCAAAGAGC
	RT-LOG4-R	GACAGGATCAAGCCAGCAAA
Solyc10g079870	RT-CKX7-F	CAGGAAGAGTGGAAGCATCAT
	RT-CKX7-R	AATCCTCTGTCCTGGAGCAA
Solyc07g061720	RT-GA2ox4-F	CCTGCCATTACTCACTTCCC
	RT-GA2ox4-R	TGAGGTTCTTGGAGTTGGCT
Solyc07g061730	RT-GA2ox5-F	GTTGAGGCCTGCGAAGAATT
	RT-GA2ox5-R	TCGCCACTTTGTCCGATTTG
Solyc08g061130	RT-HY5-F	GATCAGAAGAGTGCCGGAGA
	RT-HY5-R	CCCTTGCTTGTTGTGCTGAT
Solyc10g086500	RT-DET2-F	ATTTACCCTCTTCGCCTCCG
	RT-DET2-R	ACAACATACCCGACCCGAAT
Solyc02g089160	RT-DWF-F	ATGAAGCGAAAGGACTGGTC
	RT-DWF-R	TGCACCCCTCATGTACTTGT

Table S4 PCR primer sequences used for vector construction of pET-32a-His-BRC1.

ID	Primer (5'-3')
32a-BRC1-BamHI-F	gccatggctgatatcggatccATGTATCCTCCAAGCAACAATAACTG
32a-BRC1-SacI-R	gcaagcttgtcgacggagctcTGTTACATTTACCAACAGATACAGGC

Table S5 Probes used in the electrophoretic mobility shift assays (EMSA).

ID	Primer (5'-3')
EMSA-BRC1-WT-F--A	TTCACTTCCAACAATACCGCTACGTACCACTACCTGCTTCCTTCCT
EMSA-BRC1-WT-R--A	AGGAAGGAAGCAGGTAGTGGTACGTAGCGGTATTGTTGGAAGTGAA
EMSA-BRC1-mu-F--A	TTCACTTCCAACAATACCGCAAAAAACCACTACCTGCTTCCTTCCT
EMSA-BRC1-mu-R--A	AGGAAGGAAGCAGGTAGTGGTTTTTTGCGGTATTGTTGGAAGTGAA
EMSA-BRC1-WT-F--B	TTATATTACATGATTTTTTTTACGTGTACACCAAAGTAGTCATGAAA
EMSA-BRC1-WT-R--B	TTTCATGACTACTTTGGTGTACACGTAAAAAAAATCATGTAATATAA
EMSA-BRC1-mu-F--B	TTATATTACATGATTTTTTTAAAAAATACACCAAAGTAGTCATGAAA
EMSA-BRC1-mu-R--B	TTTCATGACTACTTTGGTGTATTTTTTAAAAAAATCATGTAATATAA
EMSA-BRC1-WT-F--C	TTATCTAGGTTAGACAAAAGCACGTGTAGTTTCAAACGCAGTAGCC
EMSA-BRC1-WT-R--C	GGCTACTGCGTTTGAAACTACACGTGCTTTTGTCTAACCTAGATAA
EMSA-BRC1-mu-F--C	TTATCTAGGTTAGACAAAAGAAAAAATAGTTTCAAACGCAGTAGCC
EMSA-BRC1-mu-R--C	GGCTACTGCGTTTGAAACTATTTTTTCTTTTGTCTAACCTAGATAA
EMSA-LOG4-WT-F-A	AGTAATCCTATTAGAAAAATGGGCCATGGACCTTGCCTTACCTAC
EMSA-LOG4-WT-R--A	GTAGGTAAGGCAAGGTCCATGGCCCATTTTTCTAATAGGATTACT
EMSA-LOG4-mu-F--A	AGTAATCCTATTAGAAAAATAAAAAATGGACCTTGCCTTACCTAC
EMSA-LOG4-mu-R--A	GTAGGTAAGGCAAGGTCCATTTTTTATTTTTCTAATAGGATTACT
EMSA-CKX7-WT-F--A	CCCCGACATCTAAAGAGTGGGCCCAAATATCAGATAAATCAAGAACA
EMSA-CKX7-WT-R--A	TGTTCTTGATTTATCTGATATTTGGGCCCACTCTTTAGATGTCGGGG
EMSA-CKX7-mu-F--A	CCCCGACATCTAAAGAGTAAACAAAAATATCAGATAAATCAAGAACA
EMSA-CKX7-mu-R--A	TGTTCTTGATTTATCTGATATTTTTGTTTACTCTTTAGATGTCGGGG
EMSA-GA2ox4-WT-F--B	TCCCAAATTTTCGAATTATTAGGGGACCATTTTTTTTAAAAAAA
EMSA-GA2ox4-WT-R--B	TTTTTTTAAAAAAAATGGTCCCCTAATAATTCGAAAATTTGGGA
EMSA-GA2ox4-mu-F--B	TCCCAAATTTTCGAATTATTAGAAAAAAATTTTTTTTAAAAAAA

EMSA-GA2ox4-mu-R--B	TTTTTTTAAAAAAAATTTTTTTCTAATAATTCGAAAATTTGGGA
EMSA-GA2ox5-WT-F--A	CAATTGTTTTGTGGGGAAAGGGTGCCCTTTCTTTTTTATTCCAA
EMSA-GA2ox5-WT-R--A	TTGGAATAAAAAAGAAAGGGCACCCTTTCCCCACAAAACAATTG
EMSA-GA2ox5-mu-F--A	CAATTGTTTTGTGGGGAAAGAATGAACTTTCTTTTTTATTCCAA
EMSA-GA2ox5-mu-R--A	TTGGAATAAAAAAGAAAGTTCATTCTTTCCCCACAAAACAATTG
EMSA-DET2-WT-F--A	TTGATAAAATAATACAAGTCATAATTTTGTTCAACGAAAGTTAAA
EMSA-DET2-WT-R--A	TTTAACTTTCGTTGAACAAAATTATGACTTGTATTATTTTATCAA
EMSA-DET2-mu-F--A	TTGATAAAATAATACAAAAAATAATTTTGTTCAACGAAAGTTAAA
EMSA-DET2-mu-R--A	TTTAACTTTCGTTGAACAAAATTATTTTTTGTATTATTTTATCAA
EMSA-DET2-WT-F--BC	GATTTGCATAAGATTCTCGTCAACACACGTGTCCACGAGGAAAAAC
EMSA-DET2-WT-R--BC	GTTTTTCCTCGTGGACACGTGTGTTGACGAGAATCTTATGCAAATC
EMSA-DET2-mu-F--BC	GATTTGCATAAGATTCTCAAAAACAAAAAAATCCACGAGGAAAAAC
EMSA-DET2-mu-R--BC	GTTTTTCCTCGTGGATTTTTTTGTTTTTGAGAATCTTATGCAAATC
EMSA-DWF-WT-F--A	TTATAGTAAAATTAACTGTGTCACTTTAACTACATGAATAACT
EMSA-DWF-WT-R--A	AGTTATTCATGTAGTTAAAGTGACACAGTTAATTTTACTATAA
EMSA-DWF-mu-F--A	TTATAGTAAAATTAACTGTAAAACTTTAACTACATGAATAACT
EMSA-DWF-mu-R--A	AGTTATTCATGTAGTTAAAGTTTTACAGTTAATTTTACTATAA

Table S6 Primers used for the ChIP-qPCR assays.

ID	Primer (5'-3')
Chip-BRC1-F	CTCTATAGTAGTTATTTGTG
Chip-BRC1-R	TAACCACATCATCACTTT
Chip-DET2-F	AAGGGTAATTTGAAGAAA
Chip-DET2-R	GCCGCTAAAAGTACATAT
Chip-DWF-F	TGCTTTCTTAAACAAATAGC
Chip-DWF-R	TCACCGAGGGAAGTAAGT

Table S7 PCR primer sequences used for vector construction in the dual-luciferase assays.

ID	Primer (5’-3’)
SK-HY5-BamHI-F	cgctctagaactagtggatccATGCAAGAGCAAGCGACGA
SK-HY5-KPNI-R	tgatttcagcgaattggtaccCTACTTCCTCCCTTCCTGTGCA
LUC-BRC1-NotI-F	tccactagttctagagcggccgcAGAATTGTCGTTATCTAGGTTAGACAAAA
LUC-BRC1-NcoI-R	tgtttttggcgtcttccatggTTGTGGAGTAGCTGTAGTTGAGGC
LUC-LOG4-NotI-F	tccactagttctagagcggccgcCTATCAAAGACAATTCAATTTTGTATCATA
LUC-LOG4-NcoI-R	tgtttttggcgtcttccatggTTAAGTGGTCAACCACATGATTAGATAT
LUC-CKX7-NotI-F	tccactagttctagagcggccgcTTATGAAAACAGTTTTCTACGGAAATAC
LUC-CKX7-NcoI-R	tgtttttggcgtcttccatggTTTTCCAATATATGTATTGAGAAAAAAAAA
LUC-GA2ox4-NotI-F	tccactagttctagagcggcIcgcGATGATTATTTTACTGATCATCAAATAGTGA
LUC-GA2ox4-NcoI-R	tgtttttggcgtcttccatggCATAGTGTTCAATGAAACATCGTTATACT
LUC-GA2ox5-NotI-F	tccactagttctagagcggccgcATGAATACTTTCGATAAAAGAAAGTTTCA
LUC-GA2ox5-NcoI-R	tgtttttggcgtcttccatggGATGTAGAAATGTGTACCAAAATTAAATATG
LUC-DWF-HindIII-F	gtcgacggtatcgataagcttTGCCTGGTTATTGCTAGCCTCG
LUC-DWF-BamHI-R	cgctctagaactagtggatccTATTAGTTTAGCCAAGCGTGTGCT
LUC-DET2-HindIII-F	gtcgacggtatcgataagcttTTGTGTTTGTATAAAATGAGAGATATTTGG
LUC-DET2-BamHI-R	cgctctagaactagtggatccGGTGTCTGCTAGCTAATTTTGCTTT
SK-BRC1-BamHI-F	cgctctagaactagtggatccATGTATCCTCCAAGCAACAATAACTG
SK-BRC1-KPNI-R	tgatttcagcgaattggtaccTGTTACATTTACCAACAGATACAGGC

Table S8 Primers used for pAbAi-baits and AD-prey constructs.

ID	Primer (5'-3')
pGADT7-BRC1-F	gccatggaggccagtgaattcATGTATCCTCCAAGCAACAATAACTG
pGADT7-BRC1-R	cagctcgagctcgatggatccTGTTACATTTACCAACAGATACAGGC
pAbAi-LOG4-F	cttgaattcgagctcggtaccCTCACAAGTTTGTGAGCTAATAATTTAGA
pAbAi-LOG4-R	atacagagcacatgcctcgagTTAAGTGGTCAACCACATGATTAGATAT
pAbAi-CKX7-F	cttgaattcgagctcggtaccGGACAATCTAACACTCTCAGCCCC
pAbAi-CKX7-R	atacagagcacatgcctcgagTTTTCCAATATATGTATTGAGAAAAAAAAA
pAbAi-GA2ox4-F	cttgaattcgagctcggtaccTGAGTAATTGTTGCTATTAATAATAGGACA
pAbAi-GA2ox4-R	atacagagcacatgcctcgagAAATTTAGTAAACAACGTTATTCGTTATATATAG
pAbAi-GA2ox5-F	cttgaattcgagctcggtaccATGAATACTTTCGATAAAAGAAAGTTTCA
pAbAi-GA2ox5-R	atacagagcacatgcctcgagGATGTAGAAATGTGTACCAAAATTAAATATG
